# Housing environment and mental health of Europeans during the COVID-19 pandemic: a cross-country comparison

**DOI:** 10.1038/s41598-022-09316-4

**Published:** 2022-04-04

**Authors:** Amélie Keller, Jonathan Groot, Joane Matta, Feifei Bu, Tarik El Aarbaoui, Maria Melchior, Daisy Fancourt, Marie Zins, Marcel Goldberg, Anne-Marie Nybo Andersen, Naja H. Rod, Katrine Strandberg-Larsen, Tibor V. Varga

**Affiliations:** 1 Section of Epidemiology, Department of Public Health, University of Copenhagen, Bartholinsgade 6Q, 1356 Copenhagen K, Denmark; 2 Population-Based Cohorts Unit, INSERM, Paris Saclay University, UVSQ, UMS 011, Université de Paris Cité, 94800 Villejuif, France; 3 INSERM, Institut Pierre Louis d’Epidémiologie et de Santé Publique (IPLESP), Equipe de Recherche en Epidémiologie Sociale (ERES), Sorbonne Université, Paris, France; 4 EpiDermE, Université Paris Est Créteil, F-94010 Créteil, France; 5 Department of Behavioural Science and Health, University College London, 1-19 Torrington Place, London, WC1E 7HB UK; 6 Faculté de Médecine, Université de Paris Cité, 75006 Paris, France

**Keywords:** Risk factors, Disease prevention, Epidemiology, Quality of life, Public health

## Abstract

Many studies have investigated the impact of the COVID-19 pandemic on mental health. Throughout the pandemic, time spent at home increased to a great extent due to restrictive measures. Here we set out to investigate the relationship between housing conditions and the mental health of populations across European countries. We analyzed survey data collected during spring 2020 from 69,136 individuals from four cohorts from Denmark, France, and the UK. The investigated housing conditions included household density, composition, and crowding, access to outdoor facilities, dwelling type, and urbanicity. The outcomes were loneliness, anxiety, and life satisfaction. Logistic regression models were used, and results were pooled using random-effects meta-analysis. In the meta-analysis, living alone was associated with higher levels of loneliness (OR = 3.08, 95% CI 1.87–5.07), and lower life satisfaction (OR = 1.27, 95% CI 1.05–0.55), compared to living with others. Not having access to an outdoor space and household crowding were suggestively associated with worse outcomes. Living in crowded households, living alone, or lacking access to outdoor facilities may be particularly important in contributing to poor mental health during a lockdown. Addressing the observed fundamental issues related to housing conditions within society will likely have positive effects in reducing social inequalities, as well as improving preparedness for future pandemics.

## Introduction

The COVID-19 pandemic transformed the everyday lives of millions of people worldwide. Public health measures such as stay-at-home recommendations, curfews, lockdown of educational activities, and remote working were implemented in most European countries during spring 2020; however, the severity of the pandemic and the stringency of public health recommendations and societal lockdowns varied throughout the pandemic.

A rapidly growing literature has investigated the effects of the COVID-19 pandemic and its associated societal lockdowns on populations’ mental health. Most of the findings from these studies indicated that the pandemic and associated societal lockdowns and periods of social isolation contributed to increased levels of loneliness, depression, and anxiety^1–8^, while a few studies examining changes in mental health indicated that some mental health outcomes improved, stayed the same, or worsened only slightly, compared to pre-pandemic years^9–11^. Considering that a national lockdown is one of the most effective non-pharmaceutical interventions to reduce the spread of COVID-19 and related deaths^12^, it is of utmost importance to understand which factors during a lockdown may lead to adverse psychological effects. Before the COVID-19 pandemic, European citizens already spent most of their time indoors^13^, but the time spent at home increased to a great extent due to the lockdowns. Individuals’ residences became the place for most of their private, work-related, and leisure activities. Robust evidence exists on housing as a determinant of health^14,15^. Thus, it is of interest to explore and quantify the relationship between the housing environment during COVID-19 related lockdowns and mental health indicators across Europe.

A few studies have assessed whether living conditions during the COVID-19 lockdowns influenced people’s mental health. For example, access to outdoor facilities has been found to be associated with lower levels of depression, stress^16^, anxiety^17^, and improved wellbeing^18^ in different studies. Increased house surface area was positively associated with wellbeing in a French study^18^ and a high prevalence of moderate-to-severe depressive symptoms was observed amongst Italian students living in dwellings < 60 m^2^^19^. Furthermore, living alone was reported to be associated with greater loneliness^20,21^. To our knowledge, no previous studies have assessed the role of housing conditions during lockdowns on multiple mental health indicators in a multi-cohort study across Europe. Using data from four European cohorts, we examined and compared whether, and to what extent, housing conditions influenced the mental health of individuals during the first COVID-19 lockdown in spring 2020.

## Methods

### Study design

A two-stage meta-analysis^22–25^ was undertaken using individual participant data from four European cohorts. This study followed the reporting principles of the Strengthening the reporting of observational studies in epidemiology (STROBE) statement^26^.

### Participating cohorts

**Figure 1:**
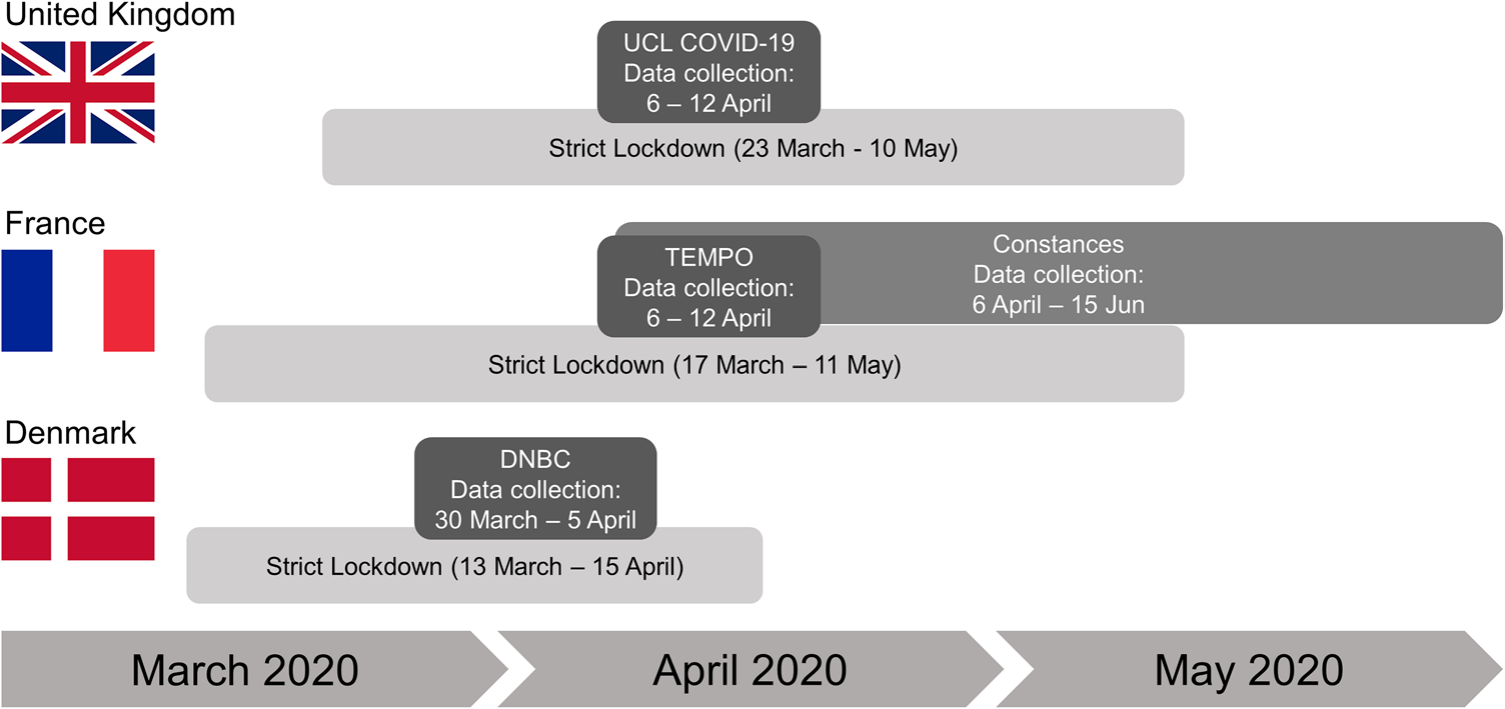
Data collection in the four participating cohorts.

Four cohorts were included in this study: The Danish National Birth Cohort (DNBC, Denmark; n = 21,889)^27^, Constances (France, n = 28,171)^28^, TEMPO (France, n = 424)^29^ and the University College London COVID-19 Social Study (UCL COVID-19, United Kingdom [UK]; n = 18,652)^20^. The participating cohorts have collected data on housing environment and mental health outcomes and are part of the international COVID-MINDS network [https://www.covidminds.org/]. The participating cohorts, relevant ethical considerations, and a sample size flowchart for all cohorts, are presented in Supplementary Text S1. The four cohorts were divided into the following age and gender categories: (1) *Young people 18–25 years*; (2) *Women above 25 years*; (3) *Men above 25 years*. Young people were considered a separate category, as young adults between ages 18 and 25 have been consistently reported to be especially vulnerable and susceptible to symptoms of poor mental health during a lockdown^30^. This category was not further stratified by gender due to sample size considerations. While some cohort surveys provided options for self-reported gender other than *men* or *women* (e.g. *other* or *prefer not to say*), the resulting groups were too small to perform analysis on. The UCL COVID-19 cohort included all three populations, while only young people and women > 25 years were included in the DNBC, and only women and men > 25 years were included in the TEMPO and Constances cohorts. All four cohorts are longitudinal; however, for this analysis, only data from the Spring 2020 lockdown period were used (March 30–April 5, 2020, for the DNBC, April 6–June 15 for Constances, April 6–April 12, for TEMPO, and April 6–April 12 for UCL COVID-19) (Fig. 1). The combined sample size for our analyses was 69,136 individuals across the four cohorts.

### Housing conditions

*Access to outdoor facilities* such as a garden, balcony, or yard was dichotomized as having access to any outdoor space (Yes or No). *Household density* was defined as the dwelling size (m^2^ floor area) divided by the number of persons living in the dwelling. The average floor area per person in the EU is 42.6 m^2^ per person^31^; hence the variable was dichotomized as < 43 m^2^ or ≥ 43 m^2^ per person. *Household crowding*^32,33^ was adapted from the Eurostat criteria^34^ with alterations due to data constraints. A minimal number of rooms for a given household was calculated as follows: (i) one room for the household; (ii) one room per couple in the household; (iii) one room for each adult single person; (iv) ½ room for each child. Household crowding was categorized as reference (minimal or minimal + 1), crowded (< minimal), and under-occupied (> minimal + 1). Based on previous literature^33^, *household composition*^35^ was categorized as households with children, adult-only households, and single-person households. *Dwelling type* was categorized as apartment versus house. In the UCL COVID-19 cohort, *urbanicity* was coded as urban (cities), semi-urban (large/small towns), and rural (village, hamlets, or other isolated dwellings) based on self-reported postal codes and EU standards of area of living^36^. In the Constances cohort, urbanicity was categorized as rural versus urban. Some of the exposures were ascertained differently across cohorts (e.g. see urbanicity definitions above); data harmonization was performed by re-categorizing survey responses to optimize the comparability of data from the four datasets.

### Indicators of mental health and wellbeing

The UCLA short three-item T-ILS version loneliness scale was used in the DNBC, TEMPO, and UCL COVID-19 to assess the degree of *loneliness* of participants. The total score ranges from 3 to 9. Scores were dichotomized, and severe loneliness was defined as scores 7–9^37^. In Constances, loneliness was measured by the question “How often have you felt lonely during the past week?”, collected on a 1–4 Likert scale from “Rarely or None of the Time (Less than 1 Day)” to “Most or All of the Time (5–7 Days)”. Scores were dichotomized, and severe loneliness was defined as score = 4.

*Anxiety* was measured using the GAD-7 scale^38^, ranging 0–21, in Constances and UCL COVID-19. Scores were dichotomized, and severe anxiety was defined as scores 15–21. In the DNBC, the SCL-ANX4^39^ was used. Answers to five questions are collected on a Likert scales ranging 0–4 and are then dichotomized to 0 (not at all) or 1 (a little-extremely) and summed for each subscale. Thus, the summed score for anxiety ranges from 0–4. Severe anxiety was defined as ≥ 3 as suggested by the authors of the CMDQ^39,40^.

The ONS Life Satisfaction scale was used to measure *life satisfaction* in the UCL COVID-19 cohort, while the Cantril Self-Anchoring Striving Scale was used in the rest of the cohorts. Both scales range between 0 (not at all satisfied with life) and 10 (completely satisfied with life). Among young people, the scale was categorized as low (0–5), medium (6–8), and high (9–10) life satisfaction according to cut-offs used in previous literature^41,42^. Among women and men > 25 years, the scale was categorized as low (0–4), medium (5–6), and high (7–10) life satisfaction according to cut-offs used in previous surveys^43^. In line with our other analysis where severe loneliness and anxiety were used as outcomes, we dichotomized life satisfaction as low vs. medium or high life satisfaction. Thus, for all three outcomes, odds ratios above 1 infer that the exposure is associated with a poorer outcome (higher loneliness and anxiety, and lower life satisfaction).

### Statistical analysis

Statistical analysis was performed using STATA for the DNBC and UCL COVID-19, SAS for Constances, and R for TEMPO.

Weighting was performed using the entropy balancing method^44^ for UCL COVID-19, and marginal calibration weighting^45^ for Constances to achieve more representative population-based samples. No weighting was performed for DNBC and TEMPO (Supplementary Text S1).

To describe the cohorts, means and standard deviations were used for normally distributed continuous variables, and medians and interquartile ranges (IQR) were used for non-normally distributed continuous variables. Frequencies and percentages were used for categorical variables.

Outcomes were used as binary variables, and binary logistic regression analyses were performed for each exposure and outcome combination within each cohort. Odds ratios (OR) and 95% confidence intervals (CI) are reported. Missing data (19.7%) was imputed using *proc mi* (SAS) in the Constances cohort. In all other cohorts, complete case analysis was performed, excluding individuals with information missing on any covariate (19% for DNBC, 43.5% for TEMPO, and 53% for UCL COVID-19). The analyses were performed separately for young people, women > 25 years, and men > 25 years. Confounders were identified using directed acyclic graphs (DAGs) and previous literature. Four models were tested:

(i) Unadjusted model: testing the association between exposure (access to outdoor facilities, household density, crowding, and composition, dwelling type, urbanicity) and outcome (high levels of loneliness, anxiety, and low levels of life satisfaction);
(ii) Model 1: Unadjusted model + age + educational attainment + sex (in the models for young people).
(iii) Model 2: Model 1 + chronic disease status + mental illness status.
(iv) Model 3: Model 2 + mutual adjustments for exposures

Age was defined as < 20 or 20-25 for young people and 25–34/35–44/45–54/55–64/65+ for women and men > 25. Educational attainment was categorized as Lower secondary education, ISCED 0–2; Upper secondary education, ISCED 3–4; Tertiary education, ISCED 5–8; Other education; Not currently studying. Chronic disease status and mental illness status were defined as Yes (ever had an illness) or No.

Results from the cohorts for each exposure-outcome combination using Model 3 were pooled using random-effects meta-analysis (*metafor* package in R), as heterogeneity was expected between the cohorts due to geographical differences, varying governmental restrictions, lockdown severities, and varying exposure and outcome ascertainment. Subgroup analyses by gender and age categories were undertaken. Subgroups analyses by country were also evaluated to investigate cross-country differences.

### Ethics statement

Ethics statements from each participating cohorts are shown in Supplementary Text S1 in detail. All experimental protocols were approved by the Danish Data Protection Agency (DNBC, Denmark), the Committee on Health Research Ethics (DNBC, Denmark), the Department of Public Health at the University of Copenhagen (DNBC, Denmark), the French Data Protection Authority (Constances, France), the institutional review board of the National Institute for Medical Research (Constances, France), the Inserm ethics committee (Constances, France), the French National Committee for Data Protection (TEMPO, France), and the UCL Research Ethics Committee (UCL COVID-19 Social Study, UK). All methods were carried out in accordance with relevant guidelines and regulations. Informed consent was obtained from all participants.

## Results

Descriptive statistics of the four cohorts can be found in Supplementary Table S1. All association results from the cohorts using the unadjusted model, Model 1, Model 2, and Model 3 (the fully adjusted model) are presented in Supplementary Table S2, Supplementary Table S3, Supplementary Table S4, and Supplementary Table S5, respectively. Forest plots showing the results from the random-effects meta-analyses for all exposure-outcome pairs, using Model 3, are shown in Supplementary Text S2. Below we present pooled and cohort-specific results using Model 3.

### Access to outdoor space

**Figure 2:**
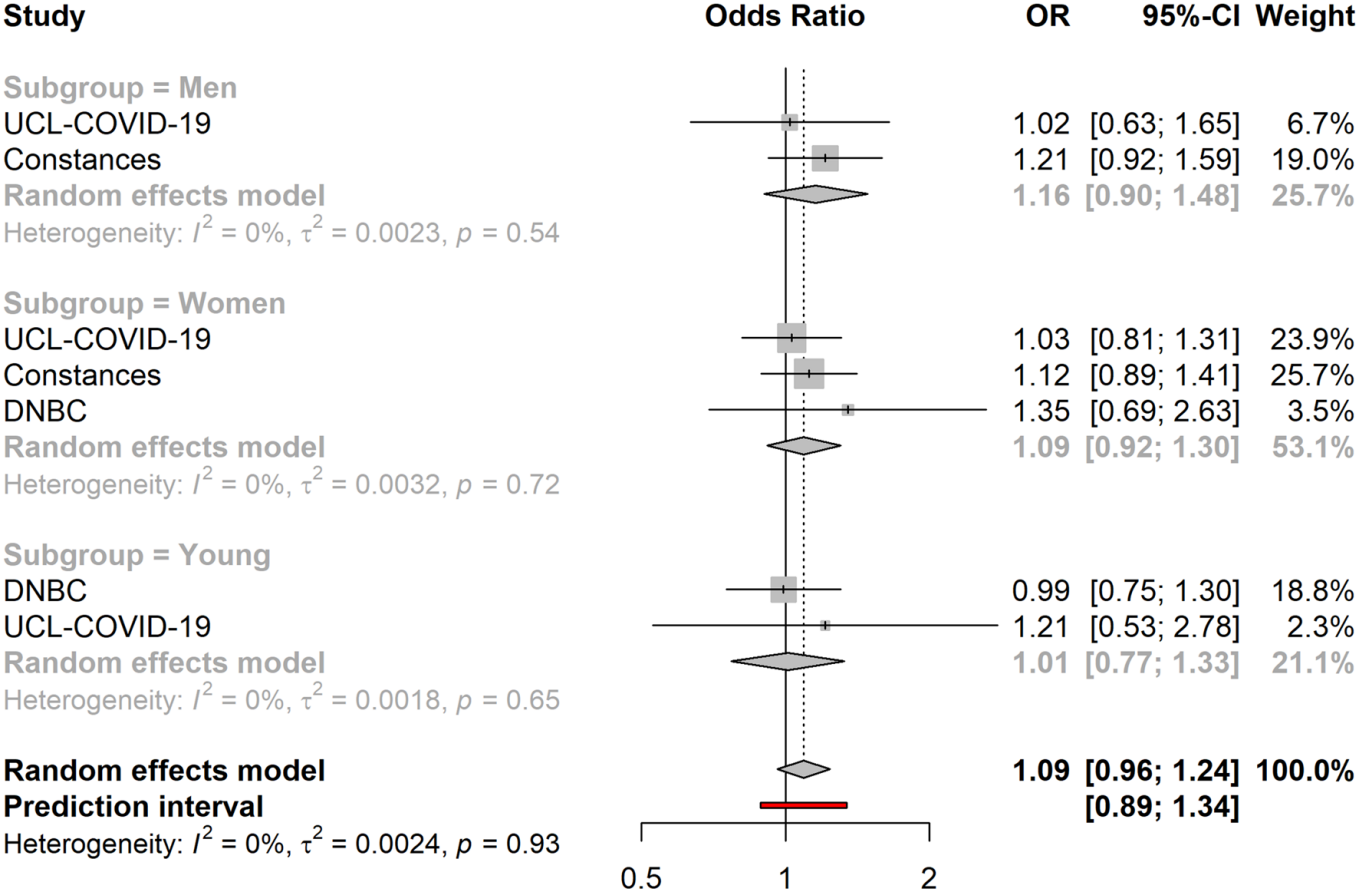
Association between access to outdoor space (yes vs. no) and high levels of anxiety (N = 68,712). The cohort-specific estimates and 95% confidence intervals were obtained using binary logistic regression models. Age, educational attainment, gender, previously diagnosed chronic disease status and presence of previously diagnosed mental illness were used a covariates. In addition, the models included mutual adjustment for the other housing exposures. *95% CI* 95% confidence interval, *OR* odds ratio.

Across all cohorts, not having access to outdoor space showed a suggestive association with higher levels of anxiety (OR = 1.09, 95% CI 0.96–1.24) (Fig. 2). Associations between access to outdoor space and loneliness (OR = 1.21, 95% CI 0.72–2.02) or life satisfaction (OR = 0.97, 95% CI 0.57–1.65) were not observed in the meta-analyses.

### Household density, crowding, and composition

**Figure 3:**
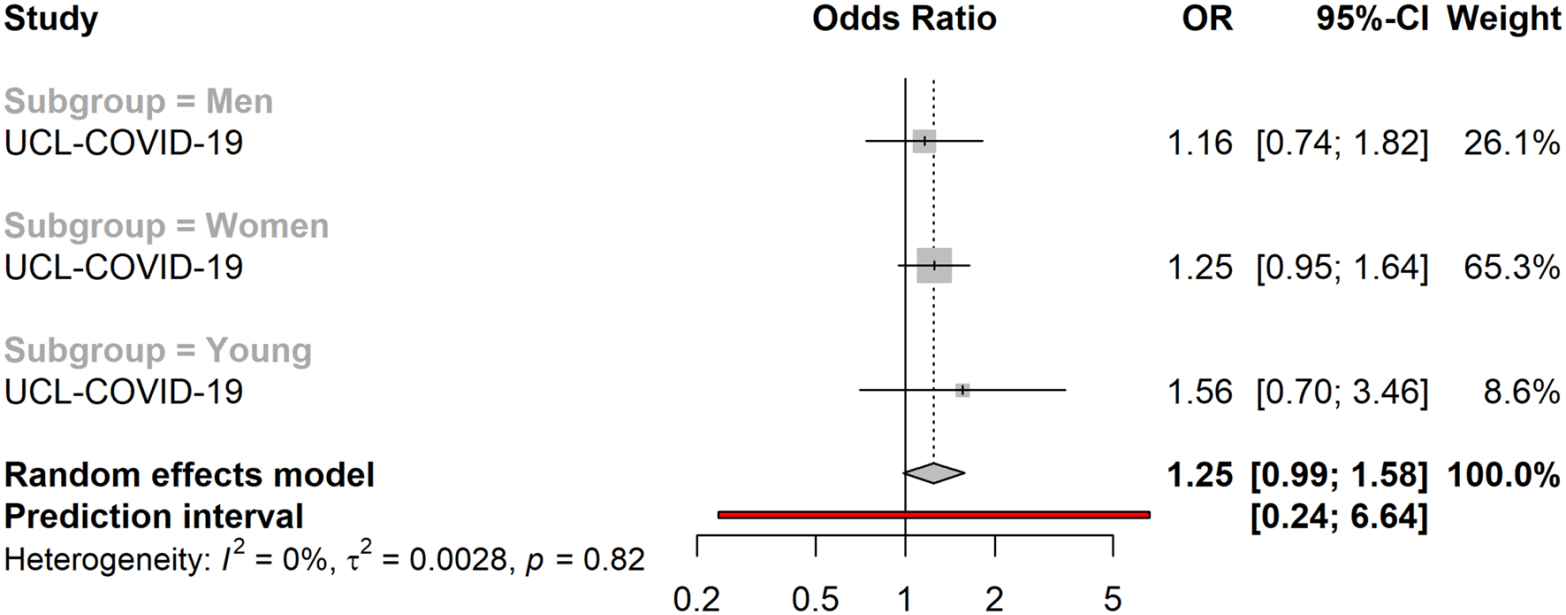
Association between household crowding and low life satisfaction (N = 18,652). The cohort-specific estimates and 95% confidence intervals were obtained using binary logistic regression models. Age, educational attainment, gender, previously diagnosed chronic disease status and presence of previously diagnosed mental illness were used a covariates. In addition, the models included mutual adjustment for the other housing exposures. *95% CI* 95% confidence interval, *OR* odds ratio.

**Figure 4:**
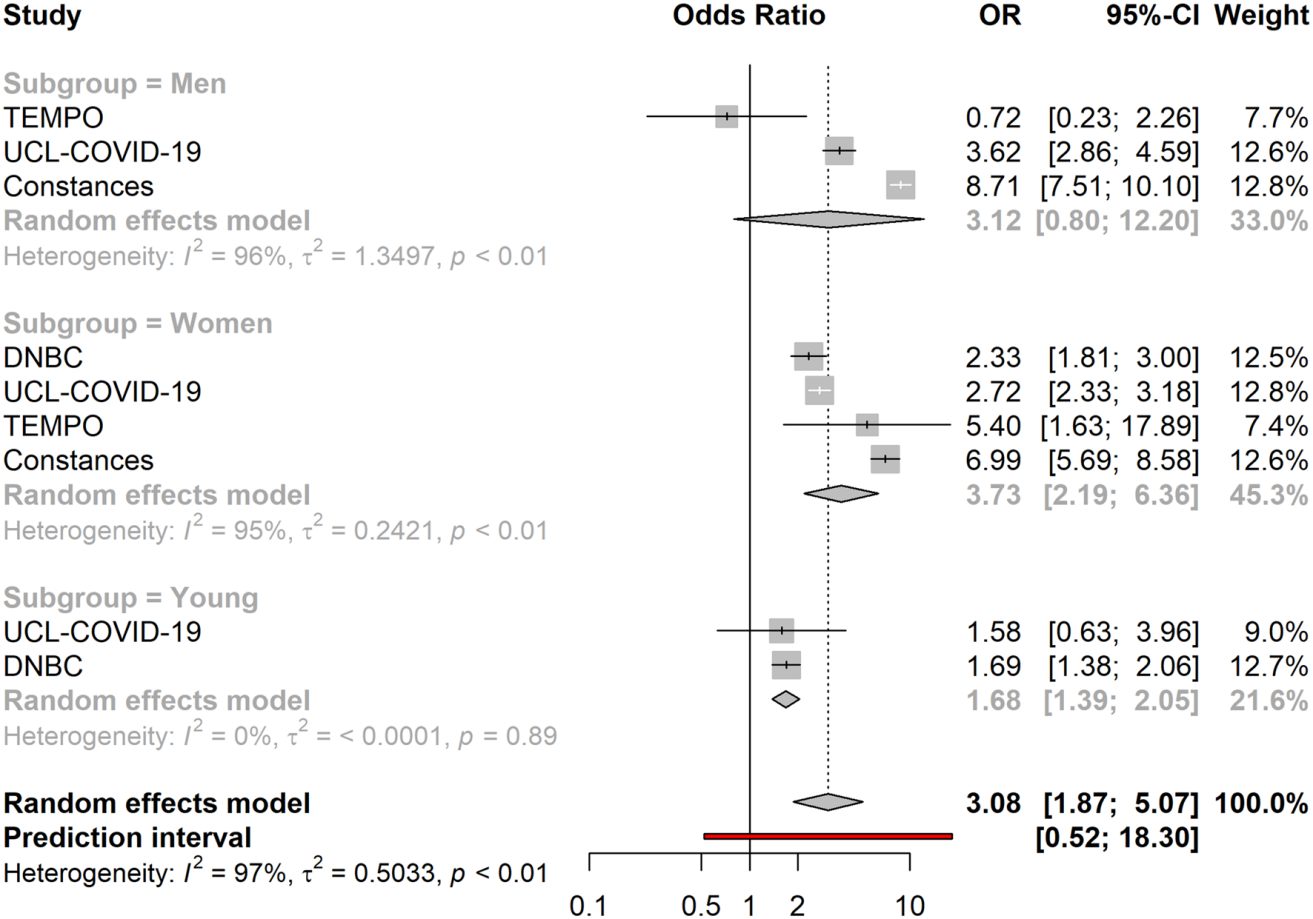
Association between living alone (vs. living with others) and high levels of loneliness (N = 69,136). The cohort-specific estimates and 95% confidence intervals were obtained using binary logistic regression models. Age, educational attainment, gender, previously diagnosed chronic disease status and presence of previously diagnosed mental illness were used a covariates. In addition, the models included mutual adjustment for the other housing exposures. *95% CI* 95% confidence interval, *OR* odds ratio.

**Figure 5:**
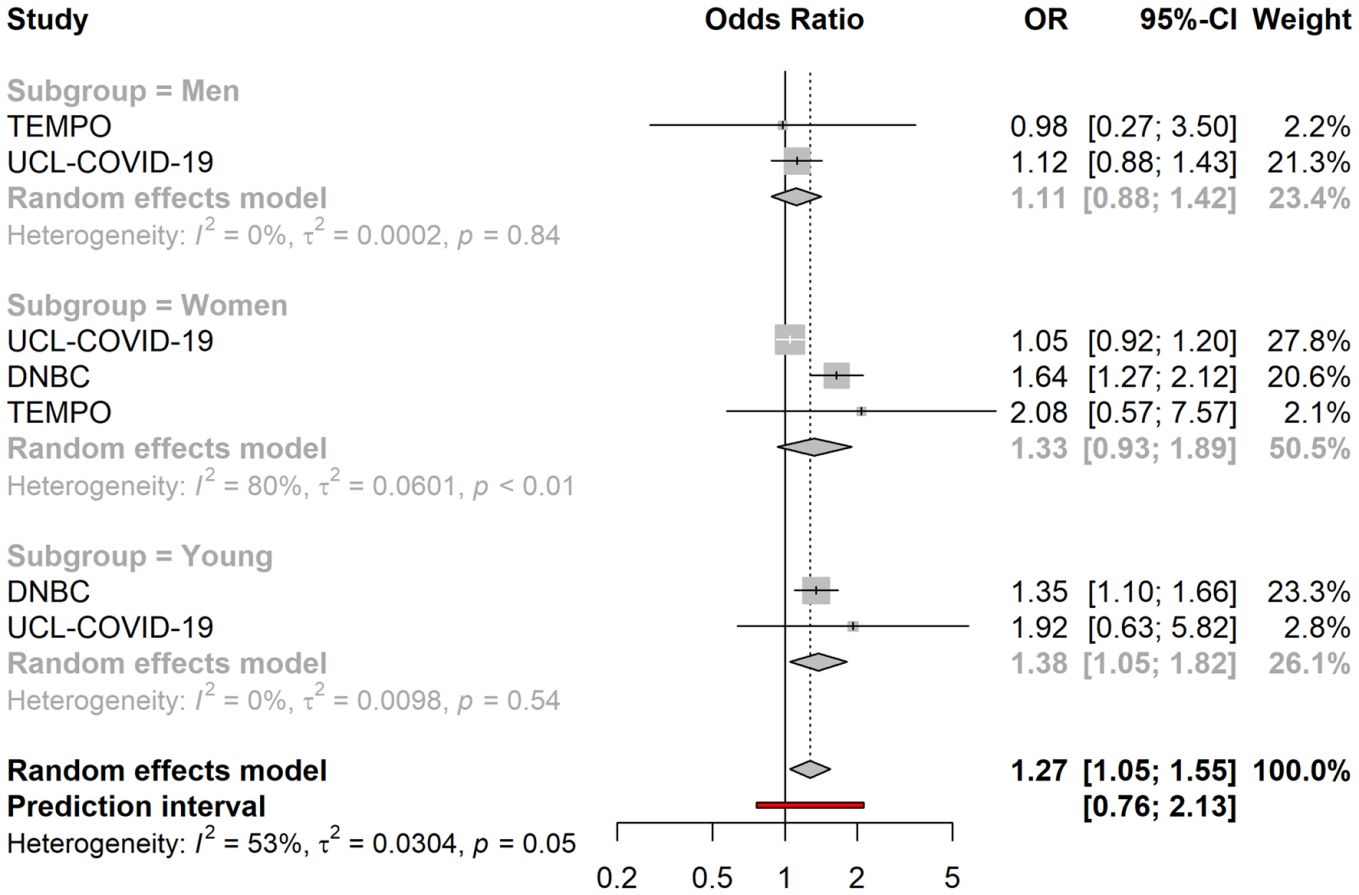
Association between living alone (vs. living with others) and low life satisfaction (N = 40,965). The cohort-specific estimates and 95% confidence intervals were obtained using binary logistic regression models. Age, educational attainment, gender, previously diagnosed chronic disease status and presence of previously diagnosed mental illness were used a covariates. In addition, the models included mutual adjustment for the other housing exposures. *95% CI* 95% confidence interval, *OR* odds ratio.

Living in under-occupied dwellings was associated with lower overall levels of loneliness (OR = 0.82, 95% CI 0.72–0.94). Similarly, living in crowded dwellings was suggestively associated with lower life satisfaction (OR = 1.25, 95% CI 0.99–1.58) (Fig. 3) and higher levels of loneliness in adult men (OR = 1.27, 95% CI 0.96–1.69) and women (OR = 1.26, 95% CI 1.01–1.58), while it was associated with lower levels of loneliness amongst young people (OR = 0.42, 95%CI: 0.21–0.84). Subgroup analyses showed that low household density, defined as having access to ≥ 43 m^2^ per person, was associated with higher levels of loneliness in men (OR = 1.36, 95% CI 1.18–1.57) and women (OR = 1.23, 95% CI 1.04–1.46) > 25 years, but lower levels of loneliness amongst the young (OR = 0.89, 95% CI 0.79–1.00). Similarly, low household density was associated with lower levels of anxiety amongst the young (OR = 0.76, 95% CI 0.67–0.86). However, living in under-occupied dwellings was associated with higher levels of anxiety in the young (OR = 2.36, 95% CI 1.08–5.17), while it was associated with lower levels of anxiety in adult men (OR = 0.77, 95% CI 0.56–1.04) and women (OR = 0.80, 95% CI 0.71–0.89). Living alone (vs. living with others) was associated with high overall levels of loneliness (OR = 3.08, 95% CI 1.87–5.07), (Fig. 4) and overall lower life satisfaction (OR = 1.27, 95% CI 1.05–0.55) (Fig. 5). While the association between living alone and loneliness was the association of the greatest magnitude that we observed in this study, no associations were detected between living alone and anxiety (OR = 0.86, 95% CI 0.59–1.25). Living with children was associated with higher levels of anxiety amongst women (OR = 1.10, 95% CI 1.00–1.22), but not in men (OR = 1.03, 95% CI 0.73–1.47), or the young (OR = 0.73, 95% CI 0.36–1.49).

### Urbanicity and dwelling type

Living in a rural environment (vs. urban environment) was not associated with either loneliness (OR = 1.06, 95% CI 0.81–1.38), anxiety (OR = 0.97, 95% CI 0.83–1.13), or life satisfaction (OR = 0.96, 95% CI 0.72–1.28) in the meta-analyses or individual cohort subgroups. Similarly, living in an apartment (vs. house) also did not associate with loneliness (OR = 0.90, 95% CI 0.51–1.61), anxiety (OR = 0.93, 95% CI 0.68–1.26), or life satisfaction (OR = 0.83, 95% CI 0.50–1.40) in the meta-analyses or individual cohort subgroups.

### Cross-country comparisons

For cross-country comparisons, we repeated our meta-analyses using countries as subgroups (Supplementary Text S3). These analyses revealed that low household density, defined as having access to ≥ 43 m^2^ per person, was associated with higher levels of loneliness in France (OR = 1.36, 95% CI 1.21–1.53), but not in other countries. Interestingly, living in crowded dwellings was suggestively associated with higher levels of loneliness in France (OR = 1.36, 95% CI 1.10–1.69), while living in under-occupied dwellings was associated with lower levels of loneliness (OR = 0.76, 95% CI 0.60–0.96) and anxiety (OR = 0.81, 95% CI 0.71–0.92). Having no access to outdoor spaces was suggestively associated with higher levels of anxiety in France (OR = 1.16, 95% CI 0.97–1.38), but not in the other countries. Living with children was suggestively associated with lower levels of loneliness in Denmark (OR = 0.90, 95% CI 0.81–1.00), while it tended to associate with higher levels of loneliness in other countries, especially amongst young people in the UK (OR = 2.87, 95% CI 1.39–5.92). Living with children was associated with higher levels of anxiety in France (OR = 1.15, 95% CI 1.01–1.31), but not in the other countries. Living alone was associated with lower life satisfaction in Denmark (OR = 1.47, 95% CI 1.20–1.80), and while directionally similar, this association did not reach statistical significance for other countries. Living alone was associated with lower levels of anxiety in the UK (OR = 0.50, 95% CI 0.31–0.80), whereas opposite trends were seen in Denmark (OR = 1.15, 95% CI 0.96–1.38) and France (OR = 1.18, 95% CI 0.71–1.96).

## Discussion

We show results from an analysis of ~ 70,000 individuals from three European countries with available housing environment and mental health data during the first lockdown of the COVID-19 pandemic during spring 2020. As previous research shows that Europeans spend most of their life indoors^13^, which was the case to an even greater extent during lockdowns with curfews and stay at home recommendations, we set out to quantify the associations between various facets of the housing environment and the mental health and wellbeing of Europeans during the first severe lockdown of the pandemic.

Our key findings are as follows: (1) Living alone was consistently related to worse mental health during lockdown; (2) living in dwellings offering more rooms for a given household was related to better mental health, albeit with some differences by country and age groups; (3) having access to outdoor space appeared to be associated with lower levels of anxiety; (4) urbanicity and dwelling types were not associated with mental health status; (5) women’s mental health seemed to be more strongly associated with household composition than men’s.

Our results regarding poorer mental health, especially loneliness, amongst people living alone during the lockdown are consistent with other studies^20,46^. As suggested in the literature, lockdown situations heighten already existing risk factors of loneliness, such as living alone^20^. Therefore, as both social isolation and loneliness have been shown to be associated with increased mortality, impaired quality of life, and wellbeing^47^, people living alone might benefit from community support during lockdowns. With the rise in the proportion of people living alone across age-groups^48^, we also recommend piloting screening methods to identify the most vulnerable individuals in communities (e.g. those living alone) for the prioritization of additional support.

Our results indicate that living in under-occupied dwellings is associated with better mental health outcomes: lower levels of loneliness and anxiety, and higher life satisfaction. Another finding is that living in less dense environments (having access to more square meters per person) is associated with worse outcomes amongst adults > 25, but better outcomes in young adults < 25. While these findings seem counterintuitive, they appear to be largely driven by results from the French Constances cohort, where (1) both measures of household density and crowding were simultaneously available, so we were able to capture the difference between these determinants; (2) both low household density (vs. high household density) and living in more crowded dwellings (vs. ideal levels) were associated with worse outcomes, e.g. higher levels of loneliness. As Constances was the only cohort where both household density and crowding were measured, we were unable to verify these findings in other cohorts. However, as emphasized above, overall results from the meta-analyses show that living in under-occupied dwellings is generally associated with better mental health. These results are supported by a 2018 systematic review on household crowding^49^, which concluded that crowding may be associated with poor mental health. During a lockdown, it has been reported that people living in overcrowded dwellings were more likely to report poor mental health than people living in non-overcrowded housing^50^.

Our results suggest that lacking access to outdoor spaces is associated with higher levels of anxiety, but not with loneliness or quality of life. Evidence from previous studies suggests that access to outdoor green and blue spaces is beneficial for mental health^51,52^. During lockdowns, several studies also reported that access to natural green spaces was associated with better mental health^53,54^. Although having access to natural green spaces might be most beneficial for mental health^55^, this study suggests that having access to any type of outdoor spaces (e.g. balcony, garden, or yard) compared to none, may lower levels of anxiety during a lockdown. Consistently, results from our previous study among young people in Denmark have shown that individuals without access to outdoor spaces experienced greater decline in well-being during the COVID-19 pandemic^21^. Therefore, housing and urban design strategies should ensure access to outdoor spaces for all^56^.

In this study, no differences in mental health indicators were found by urbanicity or dwelling type. The relationship between urbanicity and mental health pre-pandemic is contradictory, with some reports showing a clear benefit of rural environments^57^, but some showing no difference between urban and rural environments^58^. However, recent evidence pointed towards a higher prevalence of poor mental health indicators, such as anxiety disorders and PTSD, in urban environments^59^. In line with this review, recent cross-cohort analyses from the UK reported that urban residents had a higher risk of being lonely both before and during the pandemic^20^. Similarly, it was reported in a Scottish cross-sectional study that urban residents were more psychologically distressed during the COVID-19 pandemic compared to rural residents^60^. The discrepancies between our results and the UK studies might have arisen due to differences in urbanicity classification. It may also be that our definition was too broad to be sensitive to certain aspects of urbanicity that would truly matter during lockdowns, such as the urban physical (e.g. noise pollution) and social (e.g. social cohesion) environments^59^. In line with these considerations, we expect a greater proportion of dwellings with direct access to outdoor space to be rural or semi-urban, thus, mutual adjustment for these factors is expected to result in attenuated estimates relative to urbanicity, which we do observe for some of our results.

Previous studies have shown that women, young people, and those with young children experienced the greatest increase in mental distress during the COVID-19 pandemic^4,61^. In this study, women living in households with children reported higher levels of anxiety compared to men and younger adults living with children. This result might be explained by a salient aspect of the COVID-19 crisis, which involved large-scale closures of daycare centers and schools, resulting in children staying at home and needing to be cared for and home-schooled^62^. Previous studies have shown that parents experienced high levels of stress, anxiety and depressive symptoms due to homeschooling during COVID-19 lockdowns^63–65^. As, globally, women are still responsible for most of the unpaid care work, such as raising children^66^, we recommend the prioritization of the provision of child care, especially for young children, as well as flexible working hours and paid sick leave for parents during lockdowns.

The collected data make it possible to compare associations between housing environment exposures and mental health outcomes across three European countries. The Oxford COVID-19: Government Response Tracker was developed to rank a range of governmental policies in response to the pandemic on a score from 0 to 100 according to their stringency. According to this stringency metric, France had the most stringent governmental response with a score of 88 out of 100 compared to 80 in the UK and 72 in Denmark three weeks into the respective national lockdowns in the spring of 2020^67^. France and the UK likely had higher scores as both countries implemented curfews during this period, while more freedom of movement was allowed in Denmark. Thus, we expected that associations between certain characteristics of the household environment and negative mental health outcomes would be starker in France and the UK compared to Denmark. Our findings appear to be partly in line with this expectation, as a range of associations were most apparent in France and were likely driving some of the overall findings. The lack of differences in some of the examined associations can probably be attributed to the fact that the range of stringency levels in the three countries were still relatively close to each other; future comparisons with countries implementing less strict governmental responses—e.g. Sweden^68^—are warranted to explore the extent to which governmental stringency is relevant to the examined associations.

The importance of our results is threefold. First, our study can inform policy makers, authorities, and other stakeholders about the associations between various facets of the housing environment and mental health indicators in a period when citizens are encouraged or mandated to stay at home. Second, this study demonstrates that some living conditions (e.g. living alone, crowded households, lacking access to outdoor spaces) may be especially important to certain aspects of mental health during a lockdown; the identification of these factors might help to develop interventions, novel community-based support mechanisms and screening procedures to help those most affected by lockdowns. Third, while many of the reported inequalities have been observed before, it is likely that the COVID-19 pandemic and its lockdowns exacerbated some of these already existing associations. In the long term, this study might inform policy-makers and urban planners on these aspects with potential indications of specific requirements for different residents.

Important strengths of this study are its large sample size, the utilization of four cohorts from three European countries, and the availability of detailed harmonized information of housing environments, related factors and mental health outcomes during the first lockdown of the COVID-19 pandemic. Our sample includes individuals across a wide age range and from three European countries, enabling cross-country as well as sex- and age-specific comparisons.

The most important limitations of our study are the self-reported data on housing conditions and mental health outcomes, and that we did not have access to pre-pandemic data to undertake comparisons. Self-reported data are prone to various biases, and it is possible that these biases impacted our analysis. As complete-case analysis was used in most cohorts, missing data on exposures, outcomes or covariates might have also impacted our findings. This might be especially important regarding the mental health outcomes, where we expect those with the worst mental health to decide not to respond or underreport certain exposures; such biases would most likely attenuate findings. Another key limitation is that this study only included data from three Western and Northern European countries, and thus, our findings might not generalize to other settings. We consider cross-country comparisons suggestive and explorative; as results generally emanate from a single large cohort for all participating countries, it is hard to deduce whether larger, more comprehensive, and nationally representative samples would have produced different results. While weighting strategies were utilized in Constances and UCL COVID-19, where this was possible, TEMPO and DNBC were not designed to be representative of the general population. Last, in addition to our limitations regarding geographical generalizability, the cross-sectional nature of the data also limits our ability to generalize over time. Our results are based on self-reported data, which were collected at the beginning of the COVID-19 pandemic (spring 2020), at a time when most individuals likely experienced dynamic changes in their mental health in response to changing biological, societal, and political realities^6,30^.

In conclusion, we show that while urbanicity and household dwelling type was not associated with mental health, the other examined housing conditions, namely household composition, access to outdoor facilities, and household crowding and density, were associated with various aspects of mental health in three European countries during the first strict lockdowns of the COVID-19 pandemic, in spring 2020. Living alone, especially, was associated with severe loneliness and lower life satisfaction, but not with anxiety, while living with children was associated with higher levels of anxiety amongst women. These results pinpoint population groups that might need targeted interventions to ameliorate the negative mental health impact of the COVID-19 pandemic and its associated lockdowns.

## Supplementary Information

Supplementary Information 1.

Supplementary Information 2.

Supplementary Information 3.

Supplementary Table S1.

Supplementary Table S2.

Supplementary Table S3.

Supplementary Table S4.

Supplementary Table S5.

## Data Availability

Due to ethical reasons, investigators are not able to share individual level data. Summary data are shared in the Supplemental Material.
