# Supplementary material for: Housing environment and mental health of Europeans during the COVID-19 pandemic: a cross-country comparison
Source: Sci Rep. 2022 Apr 4;12:5612. doi: 10.1038/s41598-022-09316-4 (PMC8978496; doi:10.1038/s41598-022-09316-4)
Supplement: Supplementary file 1 — Supplementary Information 1. [file 41598_2022_9316_MOESM1_ESM.docx]

**Supplemental Text 1. Cohort Descriptions and Ethical Considerations**

**Danish National Birth Cohort (DK)**

An interdisciplinary research project ‘Standing together – at a distance: how Danes are living with the corona crisis’ was established in early March 2020, and a series of timed and harmonized online surveys were initiated on the 20th March 2020 to document the immediate effects of the Danish lockdown on mental health amongst different population groups. The Danish National Birth Cohort (DNBC) which is a Danish nationwide cohort of pregnant women, recruited from 1996 through 2002 and consisting of 100’415 pregnancies; detailed information on DNBC is available at: www.dnbc.dk. The online surveying was initiated in the DNBC between the 30th March 2020 and 2nd April 2020. Participants with an available email address / telephone number were invited. From the outset, 53,323 adolescents (aged 16–24) and 53,968 mothers were invited to participate. Data were available for 13,002 adolescents and 14,075 mothers at baseline and after excluding participants with missing data (19%), we had a final analytical sample of 21,889 individuals (9,205 adolescents and 12,684 mothers).

Respondents who completed the first questionnaire within one week were subsequently invited to participate in weekly surveys until the 14th May 2020 (and subsequently, if they responded to the second questionnaire, they were invited for the third questionnaire, etc.). This study is based on data on housing conditions and mental health from the first of the COVID-19 online questionnaires.

Ethical considerations: The DNBC is approved by the Danish Data Protection Agency (18/04608) and the Committee on Health Research Ethics (case no. (KF) 01-471/94). The DNBC participants were enrolled by informed consent. Ethical and personal data handling approval was obtained from the Department of Public Health at the University of Copenhagen (514-0497/20-3000). All survey respondents agreed to participate in the study and gave informed consent.

**Constances (FR)**

Constances is a large, population-based, prospective cohort whose recruitment began in 2012 and ended in 2019 with a total size of more than 200,000 subjects, including volunteers aged 18 to 69 years at baseline and living in 21 selected departments (administrative divisions) throughout metropolitan France, in both rural and urban settings, affiliated to the social security system. As part of the French project ‘Health, practices, relationships and social inequalities in the general population during the COVID-19 crisis (SAPRIS )’, the Constances cohort has collected data about the main epidemiological and social issues of the severe acute respiratory syndrome coronavirus 2 (SARS-CoV2) epidemic and the measures taken to combat it. From the 3 April 2020 questionnaires on changes in the state of health, social practices, living conditions and confidence in public and scientific actions to fight the epidemic was completed by approximately 63,864 individuals. Participants who answered to both questionnaires that were sent during the first lockdown and those with weightings data were selected. Imputations were conducted using proc mi in SAS to address missing data (19.7%). After imputation, the final analytical sample comprised 28,171 participants with complete data. Chronic diseases were collected using an annual self-administered follow-up questionnaire completed by participants at home, using either a web or paper-based questionnaire.

Ethical considerations: The Constances cohort study has received the authorization of the French Data Protection Authority (CNIL: Commission Nationale de l’Informatique et des Libertés) and the institutional review board of the National Institute for Medical Research (Authorization number 910486). Ethical approval and written or electronic informed consent for participation in the SAPRIS cohort were obtained from each participant before enrolment in the original cohort. The SAPRIS survey was approved by the Inserm ethics committee (approval #20-672 on March 30, 2020). All subjects included in this study gave their informed consent.

**TEMPO (FR)**

The TEMPO study is a French ongoing prospective cohort which aims to evaluate individual, familial and social determinants of mental social health and addictive behaviors. The initial cohort was established in 1991, recruiting its participants via their parents who participated in the GAZEL cohort study. A second recruitment phase took place in 2011 where all young adults whose parents participated on the GAZEL cohort study were invited. Data collection took place in 1991 (n=2,658) and 1999 (n=1,270). Parents were asked to complete a questionnaire regarding the living conditions and mental health of the participant, and the participants answered the questionnaire themselves in 1999 (n=1,148), 2009 (n=1,103), 2011 (n=1,214), 2014 (n=786), 2018 (n=864) and 2020 (8 questionnaires proposed in total). From the 24 March 2020 a questionnaire on the changes experienced in terms of health and lifestyle as a result of the lockdown due to the Covid-19 epidemic was sent the TEMPO cohort participants. The TEMPO Covid-19 cohort included 750 participants aged 26-45 years. After excluding participants with missing data (43.47%), we had a final analytical sample of 424 participants.

Ethical considerations: The TEMPO cohort was approved by the French national committee for data protection (CNIL: Commission Nationale de l’Informatique et des Libertés) (Authorization number 908163). All subjects included in this study gave their informed consent.

**UCL COVID-19 Social Study (UK)**

This study analyzed data from the UK COVID-19 Social Study run by University College London, a longitudinal study that focuses on the psychological and social experiences of adults living in the UK during the COVID-19 pandemic. The study commenced on 21st March 2020 and involves weekly online data collection from participants for the duration of the pandemic. The study is not random and therefore is not representative of the UK population. However, it does contain a well-stratified sample that was recruited using three primary approaches. First, convenience sampling was used, including promoting the study through existing networks and mailing lists (including large databases of adults who had previously consented to be involved in health research across the UK), print and digital media coverage, and social media. Second, more targeted recruitment was undertaken focusing on (i) individuals from a low-income background, (ii) individuals with no or few educational qualifications, and (iii) individuals who were unemployed. Third, the study was promoted via partnerships with third sector organizations to vulnerable groups, including adults with pre-existing mental health conditions, older adults, carers, and people experiencing domestic violence or abuse. A full protocol for the study is available online at [www.COVIDSocialStudy.org](http://www.COVIDSocialStudy.org).

The UCL analyses COVID-19 Social Study focused on the third week during the first lockdown in the UK (6^th^ April-12^th^ April 2020) (Figure 2). In total, there were 39,284 participants who responded during the observational period. After excluding participants with missing data (53%) (mostly due to missing data on outdoor variables), we had a final analytical sample of 18,652, including young people (aged 18-24) of 499, women (aged 25+) 13,592 and men (aged 25+) 4,561. Data were weighted to the proportions of sex, age, ethnicity, education, and country of living obtained from the Office for National Statistics.

Ethical considerations: The study was approved by the UCL Research Ethics Committee [12467/005] and all participants gave informed consent. All participants provided fully informed consent. The study is GDPR compliant.

**Weighting methods**

**Entropy Balancing Method** – employed in the UK COVID-19 Social Study (UK)

The cross-sectional weighting was implemented by using Stata, using the user-written package ‘ebalance’ [https://web.stanford.edu/~jhain/Paper/JSS2013.pdf]. The weighted data were matched to population statistics for the following domains: age; sex; ethnicity; educational attainment; country of living. These statistics were extracted from the Office for National Statistics

[https://www.ons.gov.uk/peoplepopulationandcommunity/populationandmigration/populationestimates/datasets/populationestimatesforukenglandandwalesscotlandandnorthernireland].

Reference for method: Hainmueller J. Entropy Balancing for Causal Effects: A Multivariate Reweighting Method to Produce Balanced Samples in Observational Studies. Political Analysis 2012; 20(1): 25–46.

**Marginal Calibration Weighting Methods** – employed in the Constances cohort (FR)

The weighting method employed relies on scores estimation that is grouped together as a homogeneous response. This method was applied to calculate the weight of participation to the questionnaire and a weight for participation request to the questionnaire. These two weights allowed for obtaining the final weight. The obtained weights are then calibrated on the margins of the target population of Constances. The calibration variables are the ones that are usually used in the calculation of the annual weightings of Constances. In total, 66,680 individuals were sent a request to participate in the Covid questionnaire (QCovid), out of which 47,326 participated. The target population of Constances included volunteers aged 18 to 69 years affiliated to the social security and living in the 21 selected departments of Constances. The method used is based on the one used to setting up the weightings in the Constances cohort. The variables used are the following: sex, age class, employment status, geographical location, affiliation to the social security.

Reference for method: Deville JC, Särndal CE. Calibration estimators in survey sampling. Journal of the American Statistical Association 1992; 87: 376-82.

**No weighting** – employed in the DNBC cohort (DK), and the TEMPO cohort (FR)

**Sample size flowchart**
