# Supplementary material for: Housing environment and mental health of Europeans during the COVID-19 pandemic: a cross-country comparison
Source: Sci Rep. 2022 Apr 4;12:5612. doi: 10.1038/s41598-022-09316-4 (PMC8978496; doi:10.1038/s41598-022-09316-4)

**Supplemental Text 2. Forest plots (age and gender stratified subgroup analysis).**

**Severe loneliness**

No access to outdoor facilities vs access to outdoor facilities


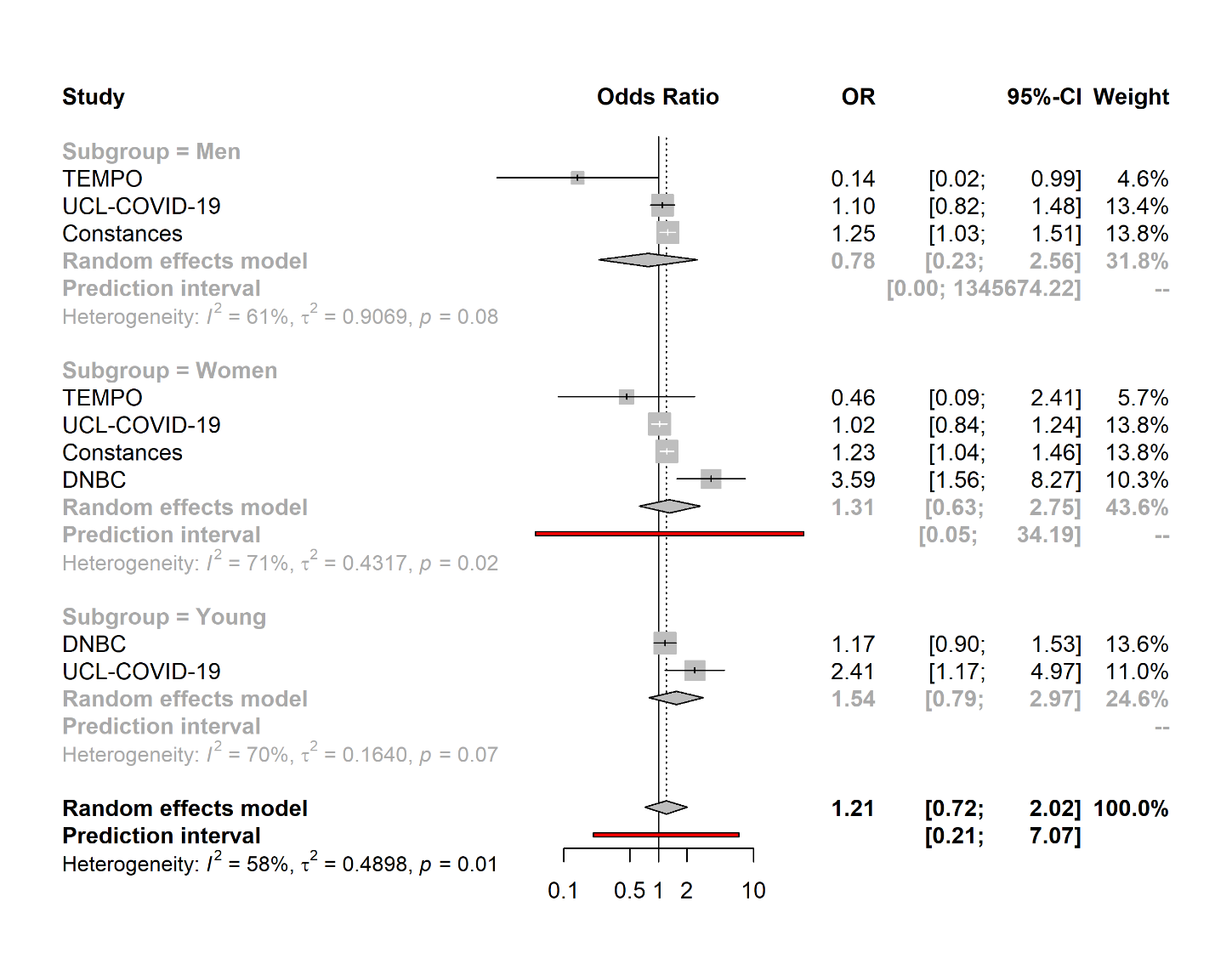


**Severe loneliness**

Household density ≥43m^2^ vs <43m^2^


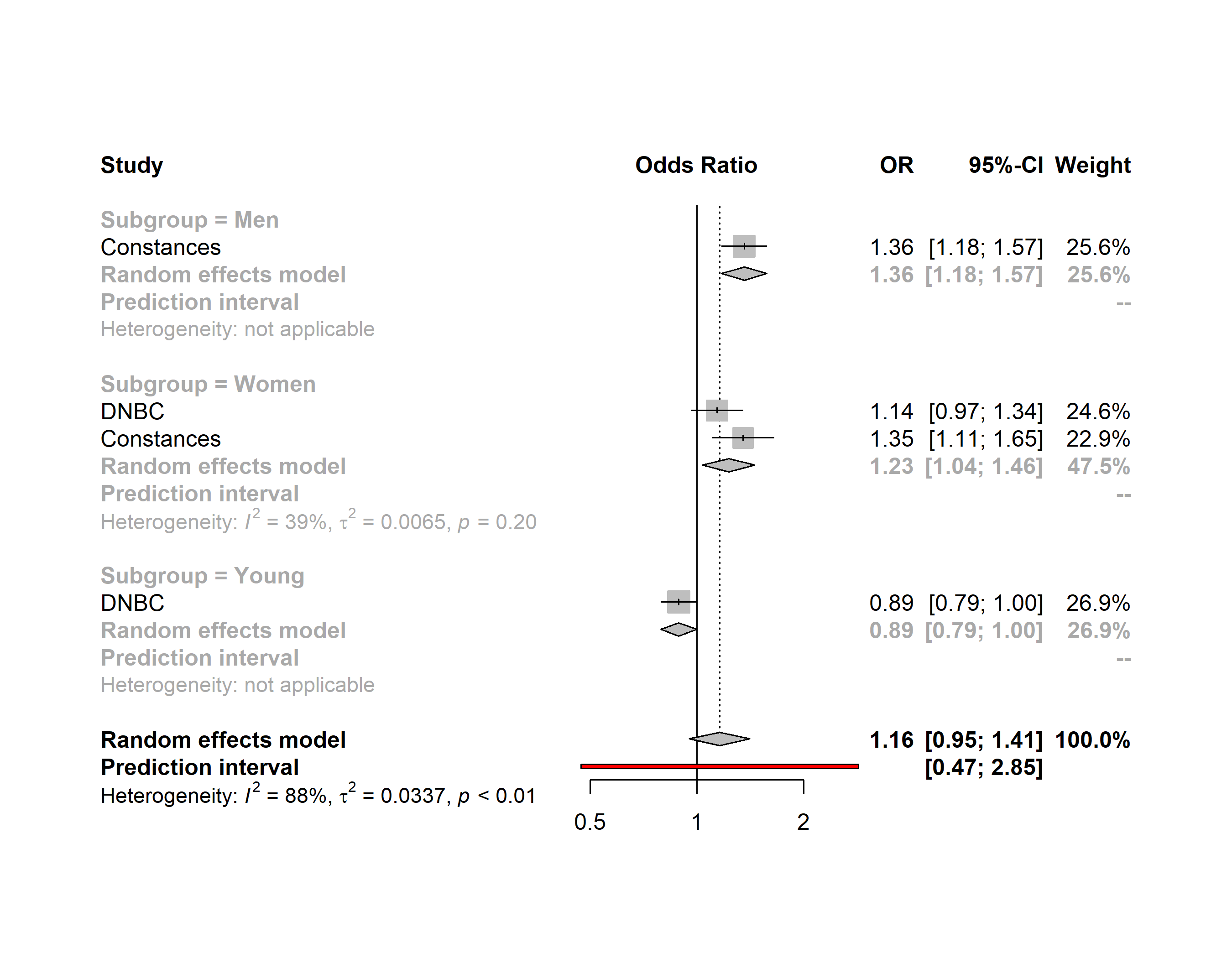


**Severe loneliness**

Household crowded vs. ideal


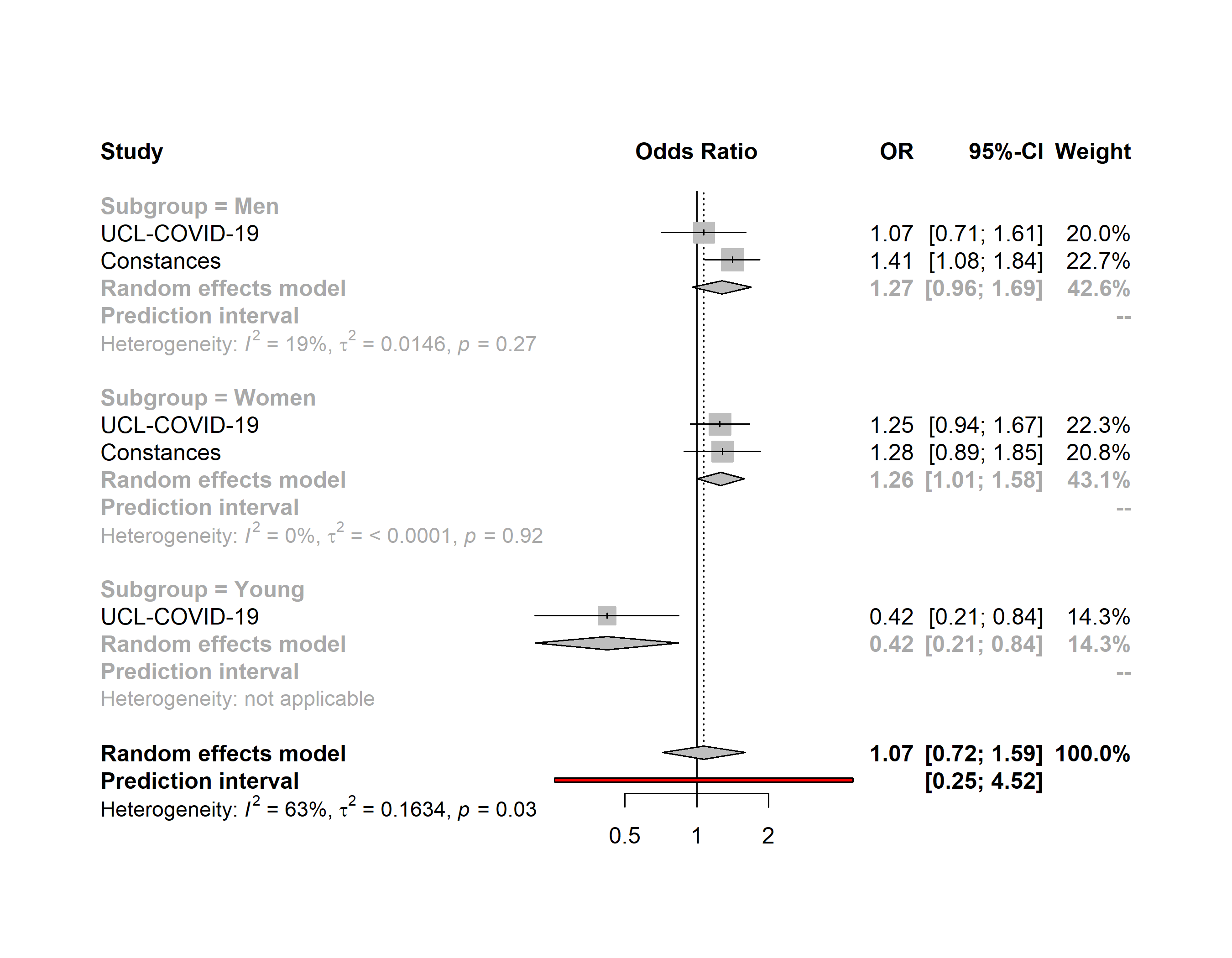


**Severe loneliness**

Household underoccupied vs. ideal


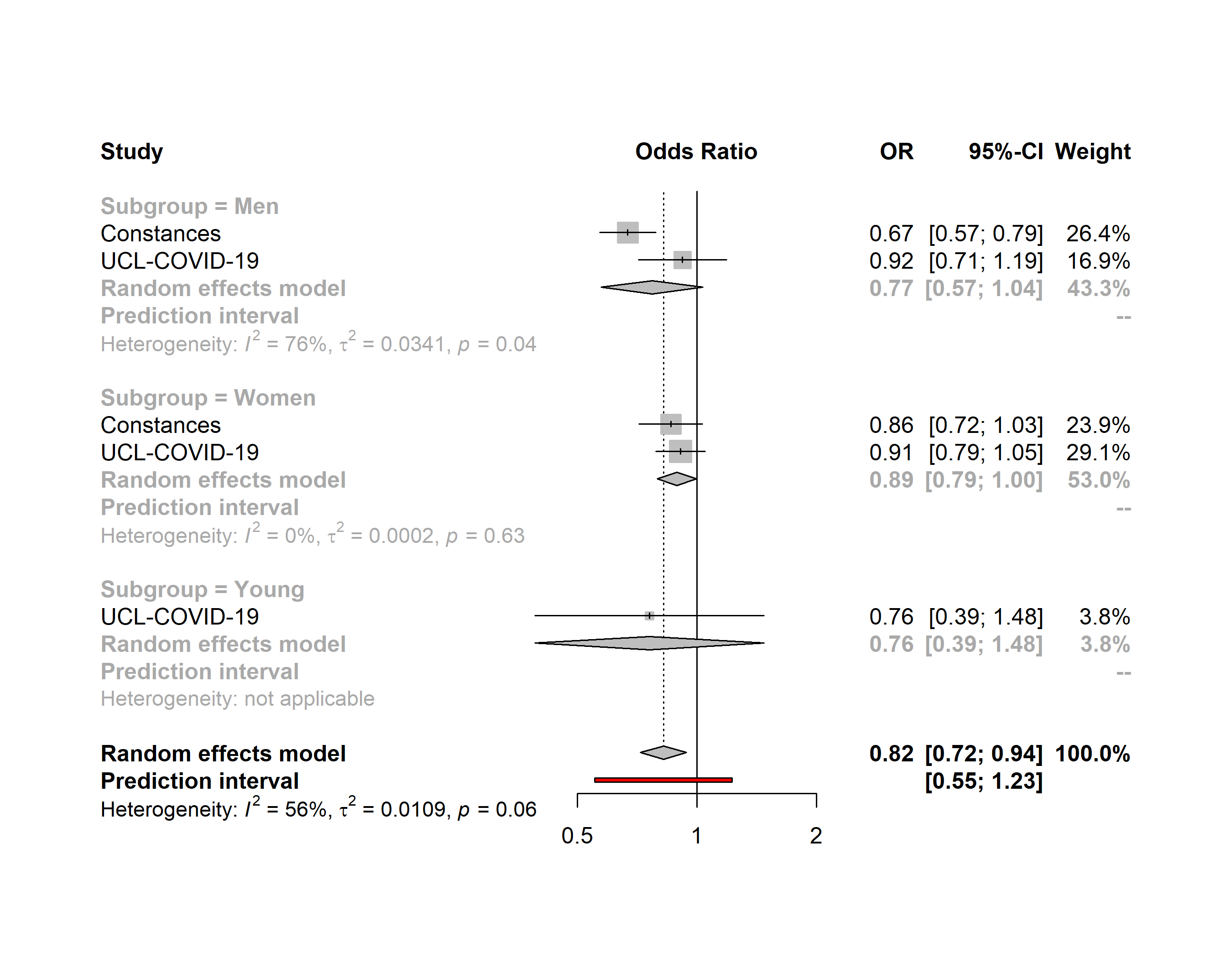


**Severe loneliness**

Households with children vs. Adults-only households


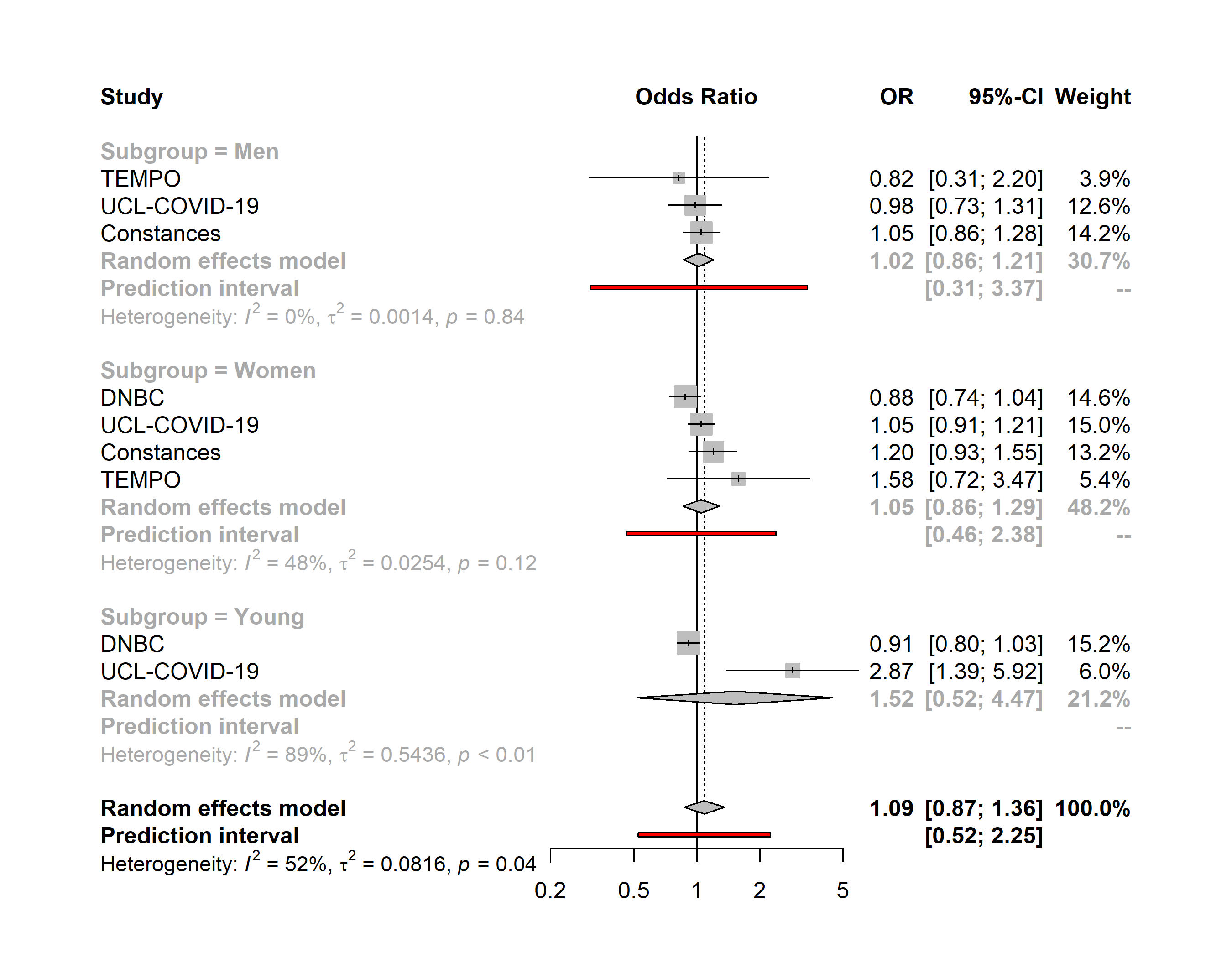


**Severe loneliness**

Living alone vs. Adults-only households


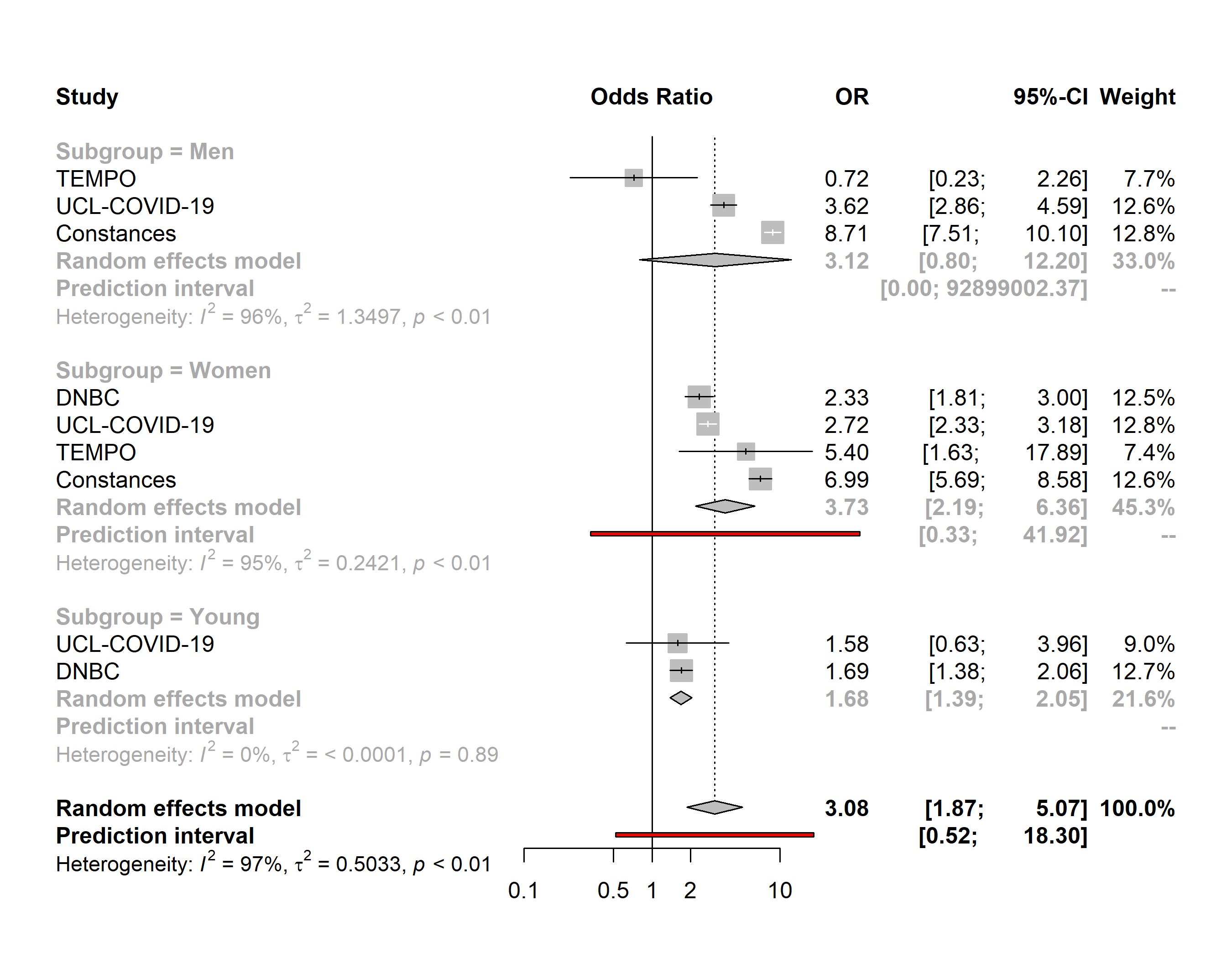


**Severe loneliness**

Apartment vs. House


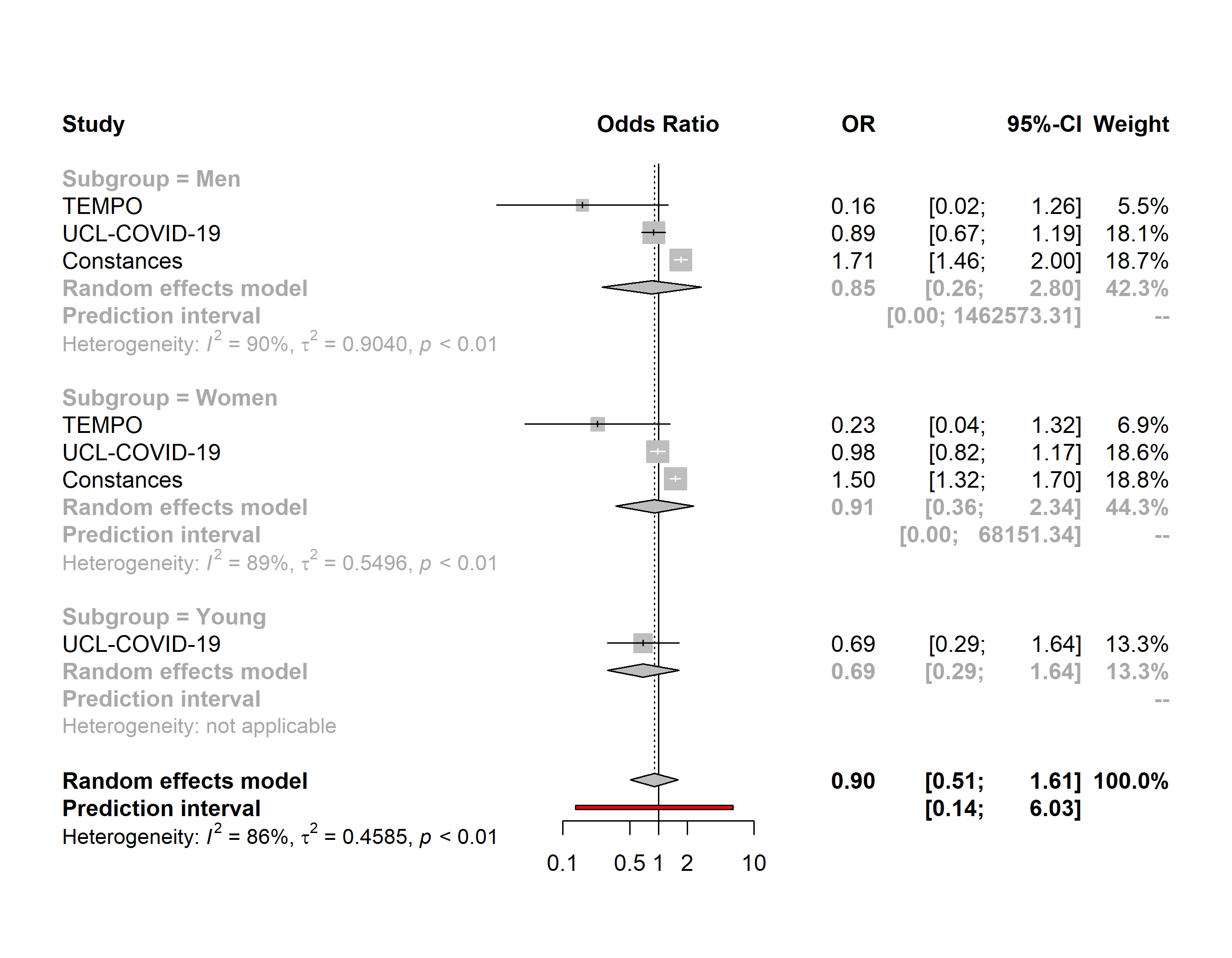


**Severe loneliness**

Semi-urban environment vs. Urban environment


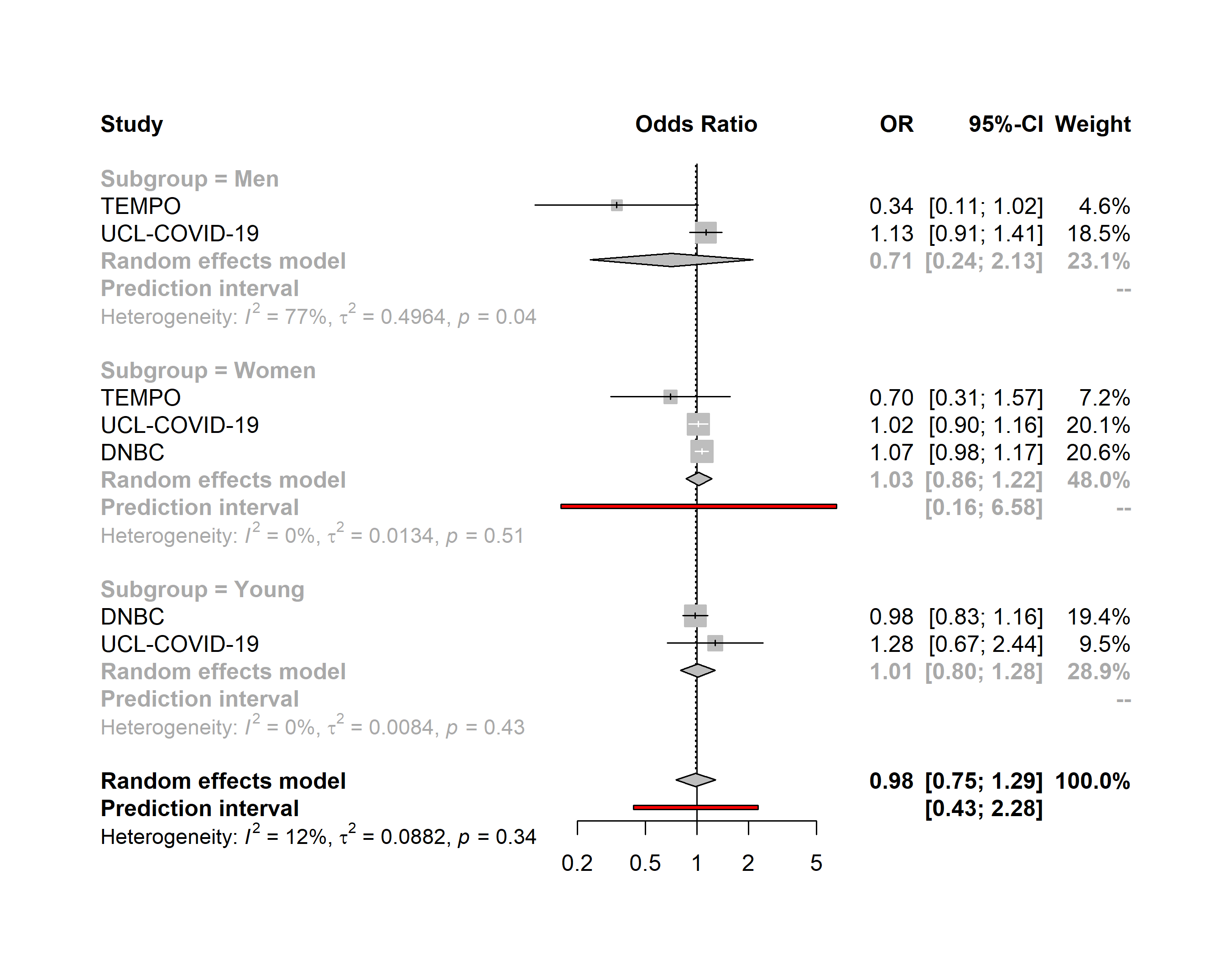


**Severe loneliness**

Rural environment vs. Urban environment


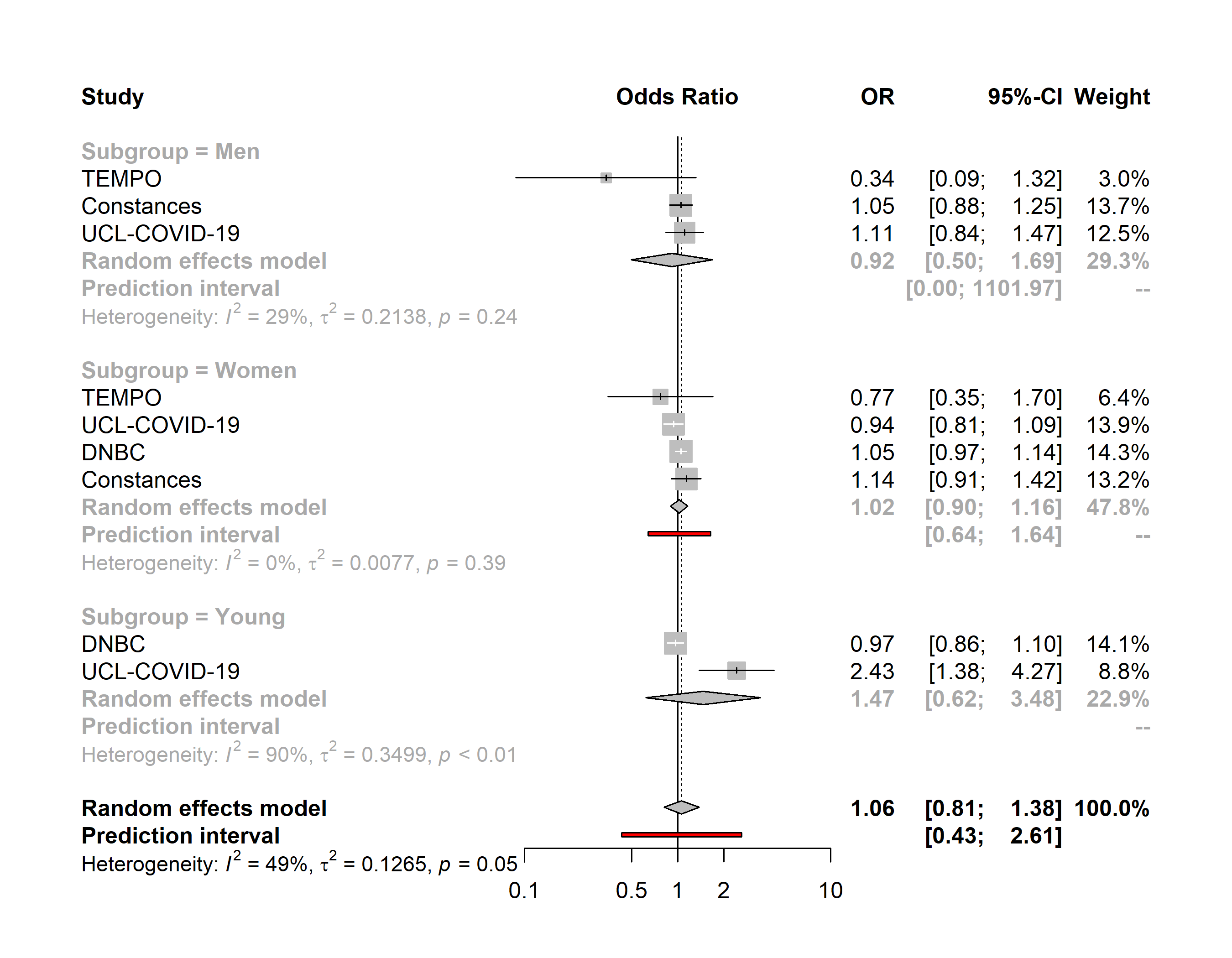


**Severe anxiety**

No access to outdoor facilities vs access to outdoor facilities


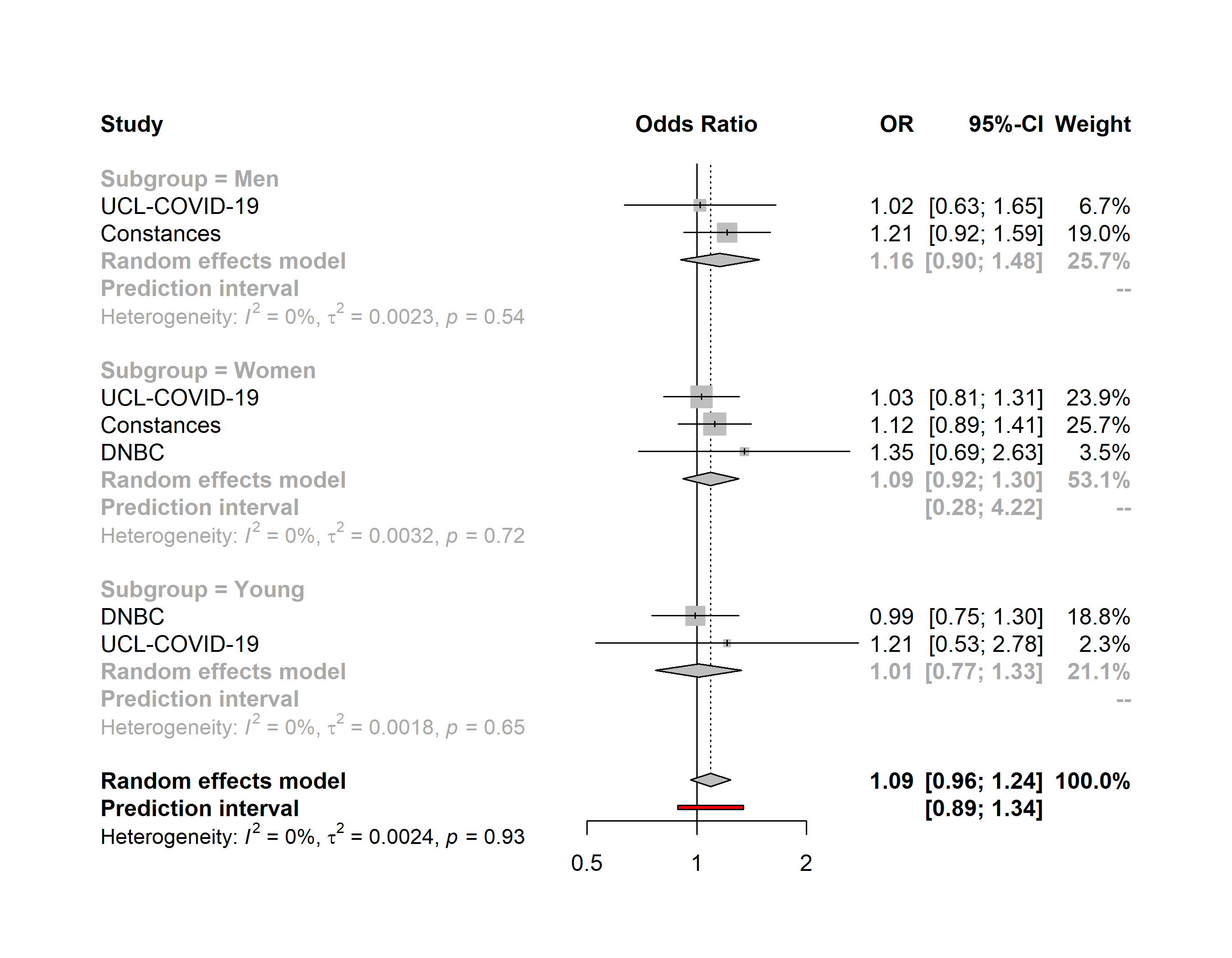


**Severe anxiety**

Household density ≥43m^2^ vs <43m^2^


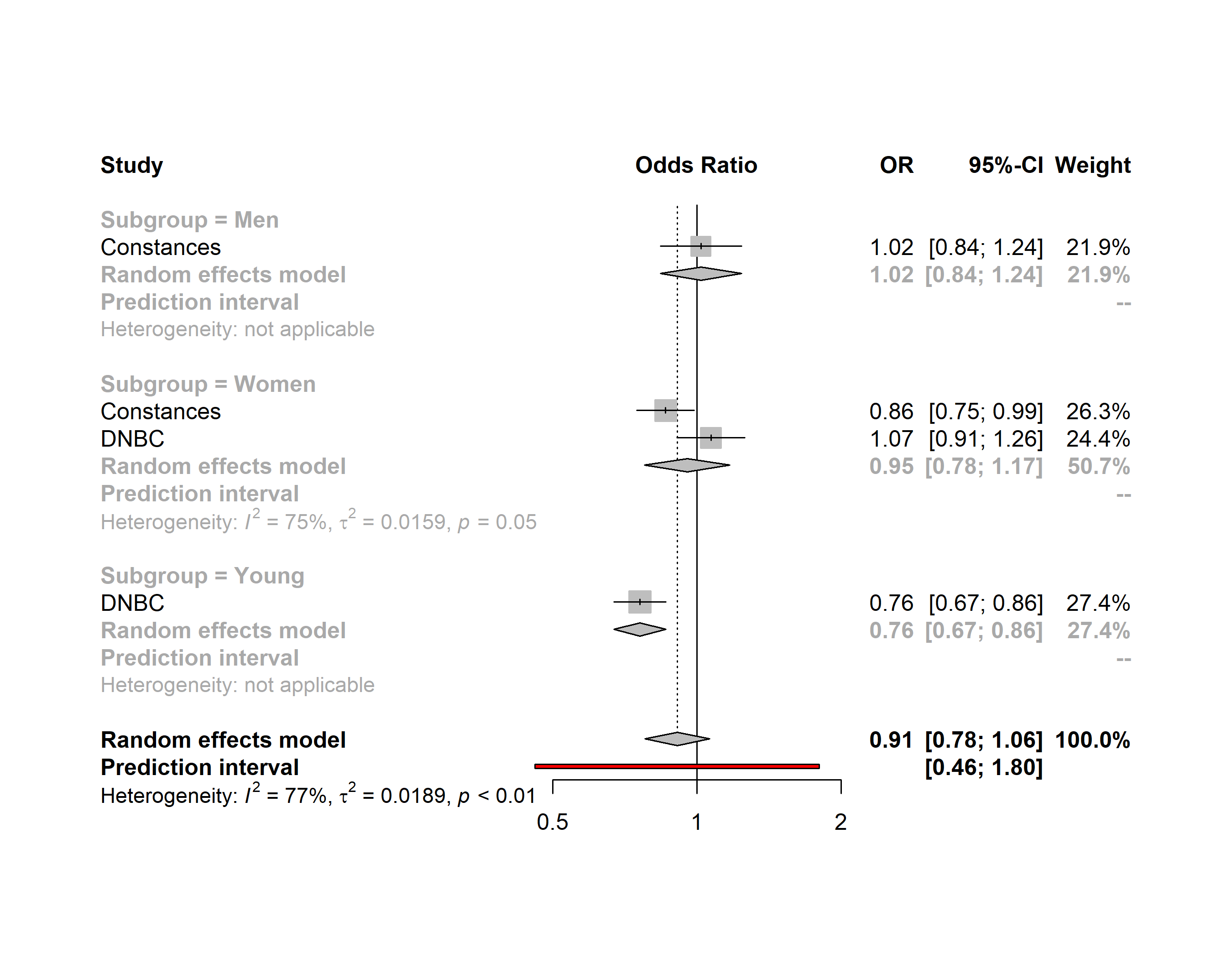


**Severe anxiety**

Household crowded vs. ideal


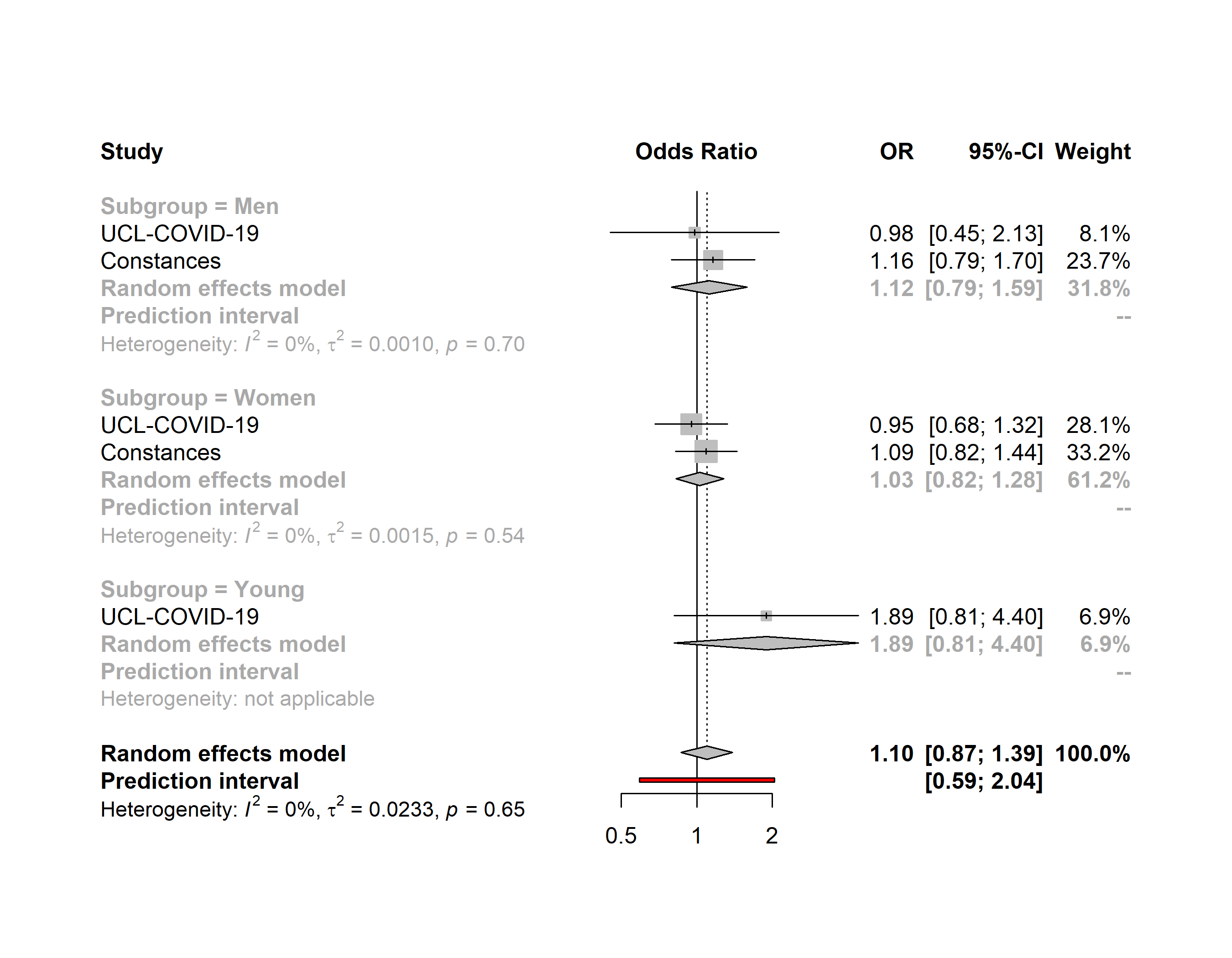


**Severe anxiety**

Household underoccupied vs. ideal


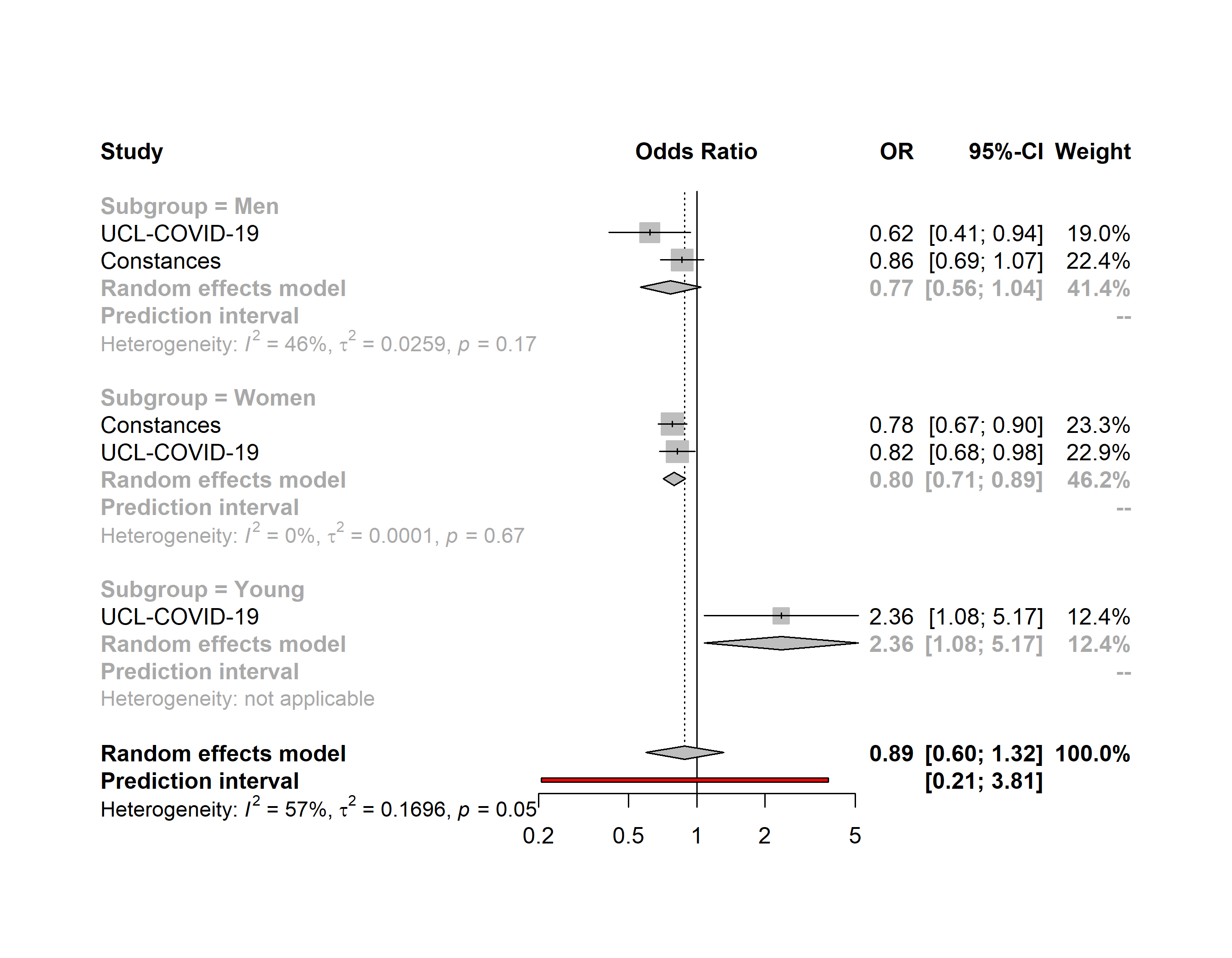


**Severe anxiety**

Households with children vs. Adults-only households


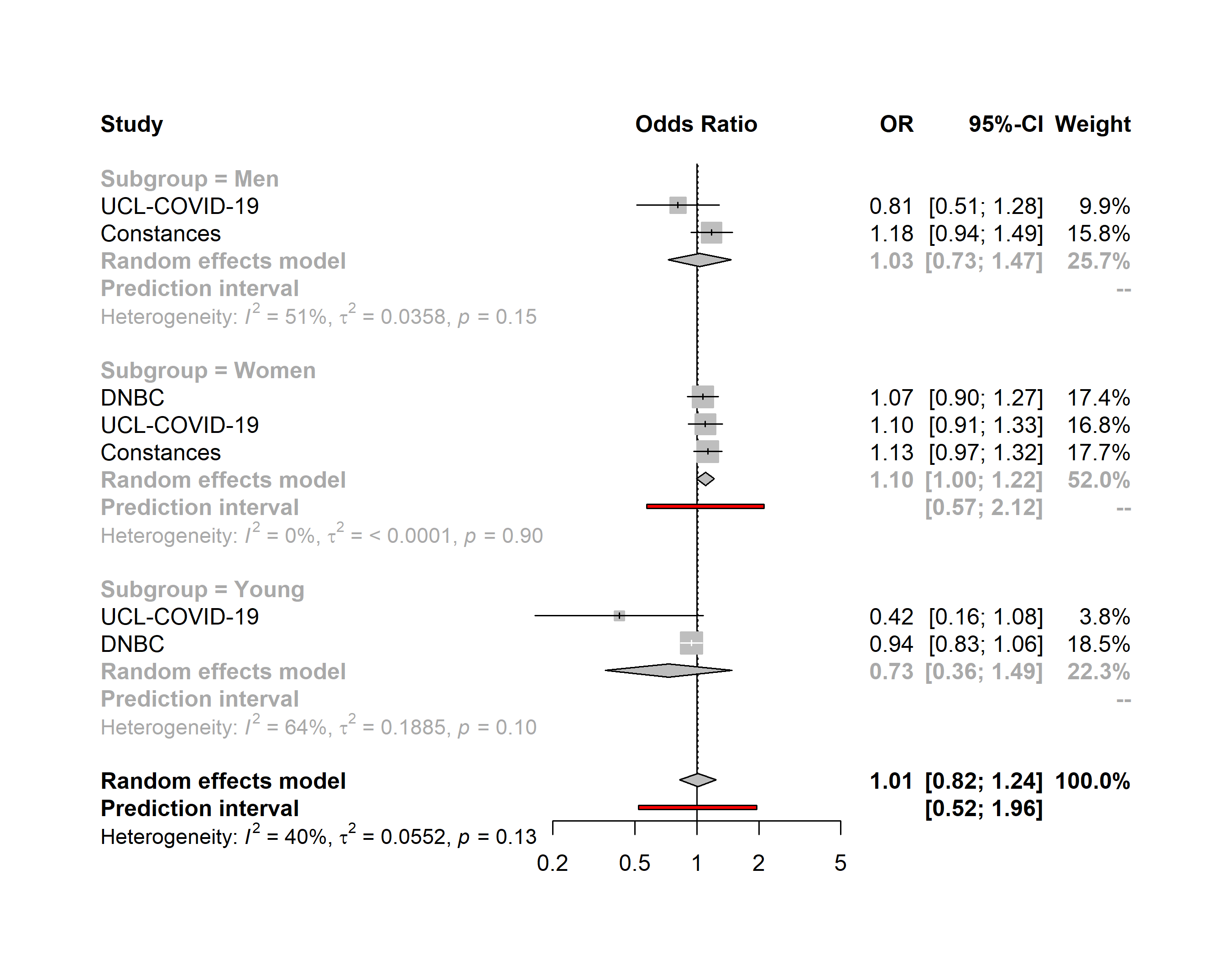


**Severe anxiety**

Living alone vs. Adults-only households


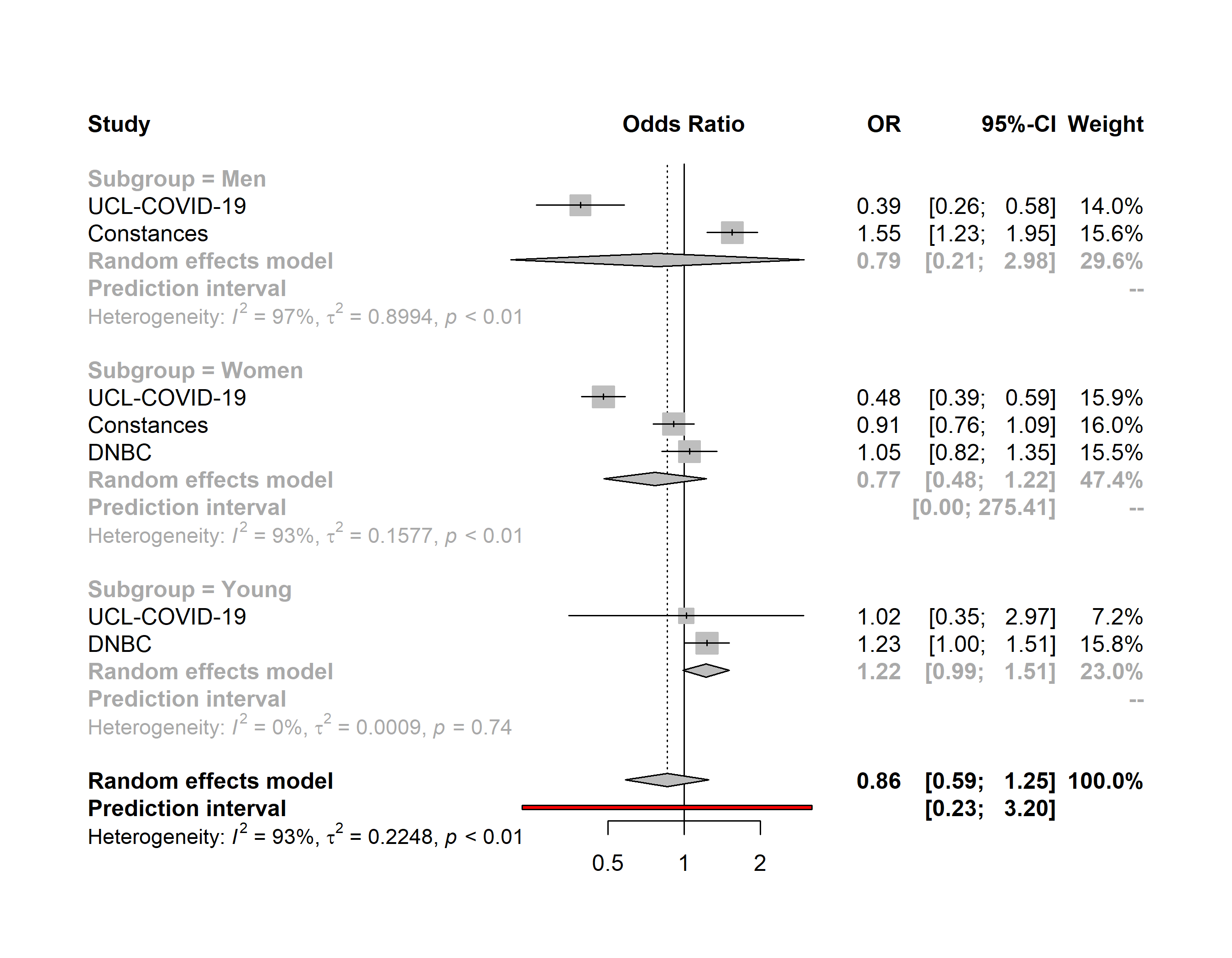


**Severe anxiety**

Apartment vs. House


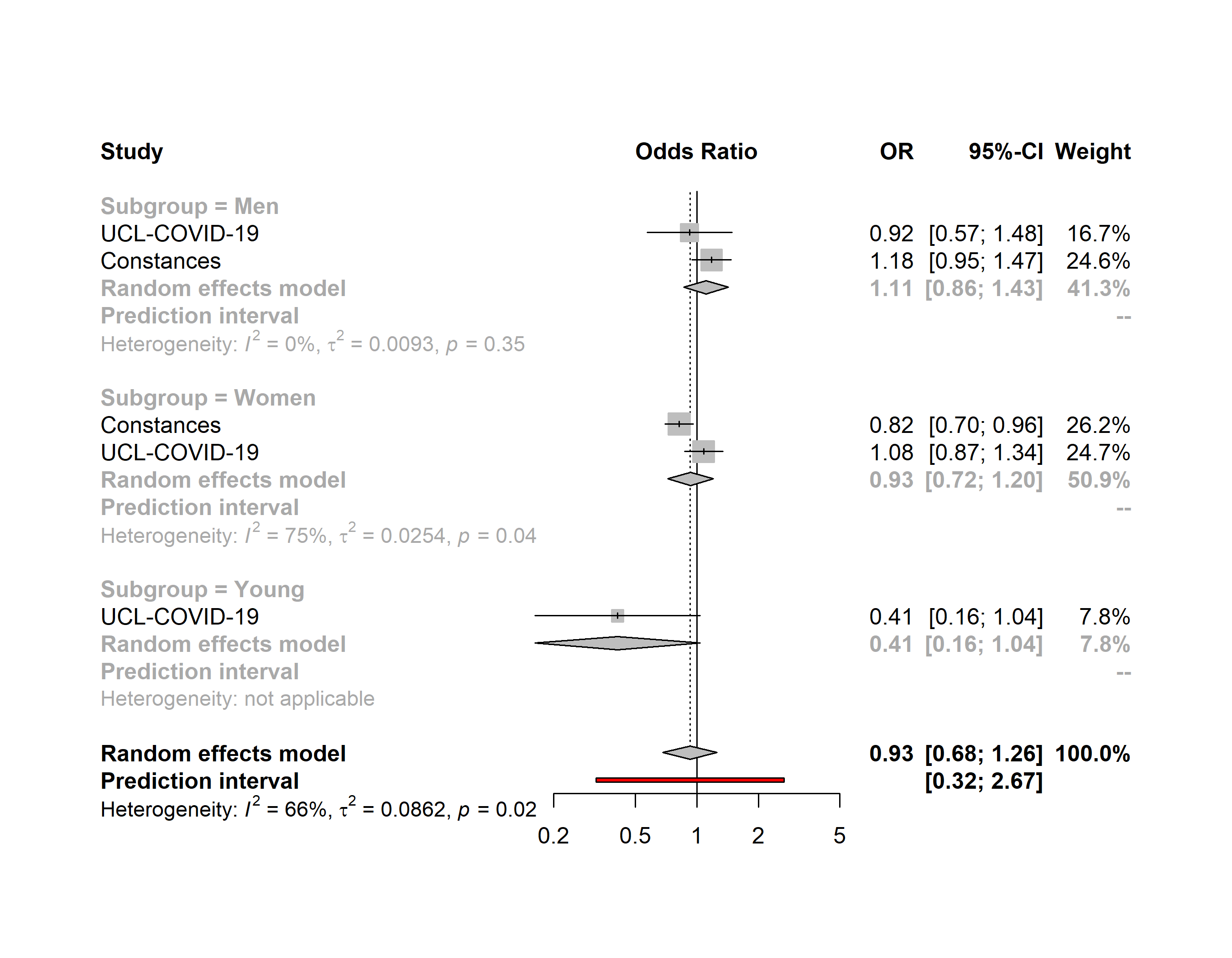


**Severe anxiety**

Semi-urban environment vs. Urban environment


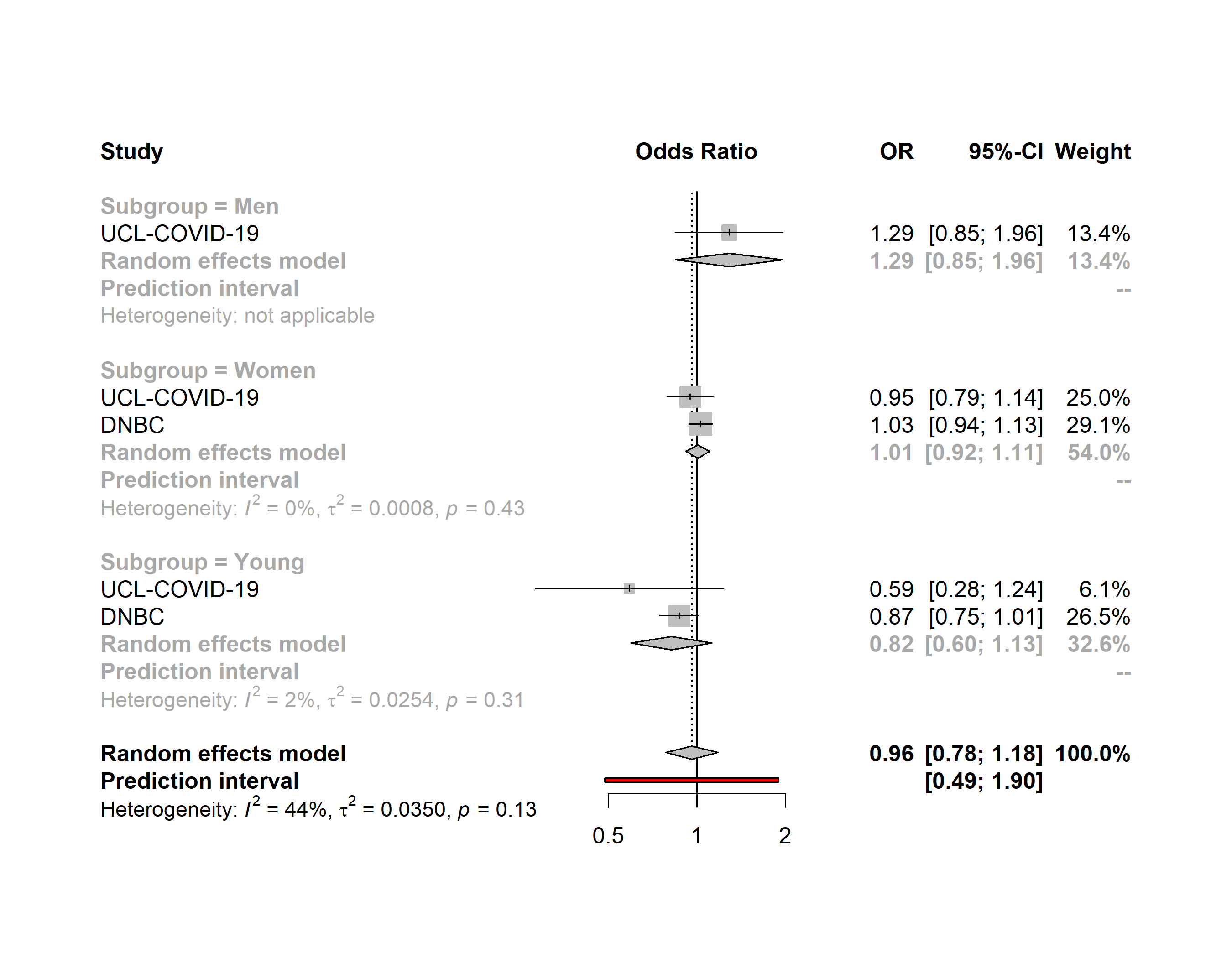


**Severe anxiety**

Rural environment vs. Urban environment


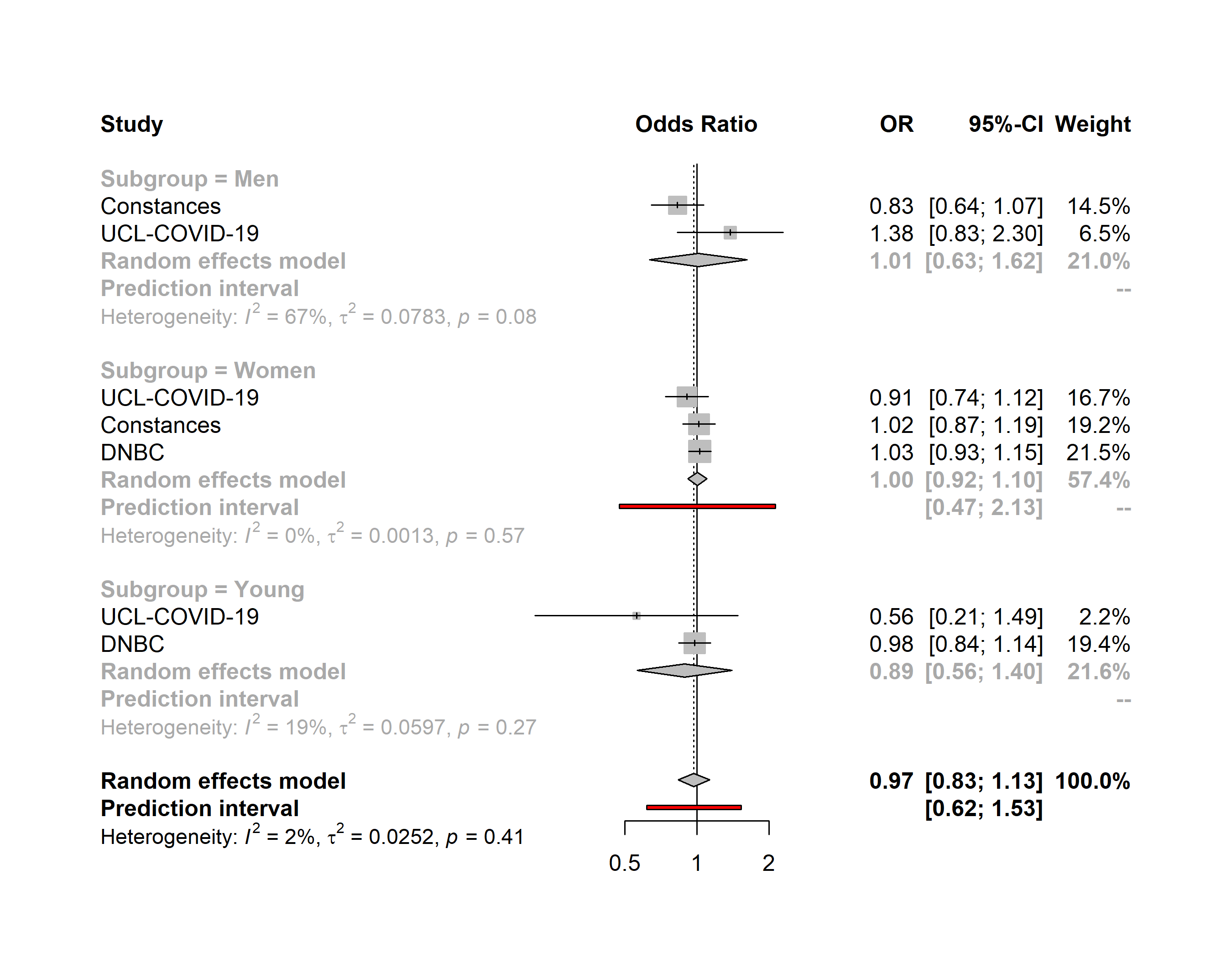


**High life satisfaction**

No access to outdoor facilities vs access to outdoor facilities


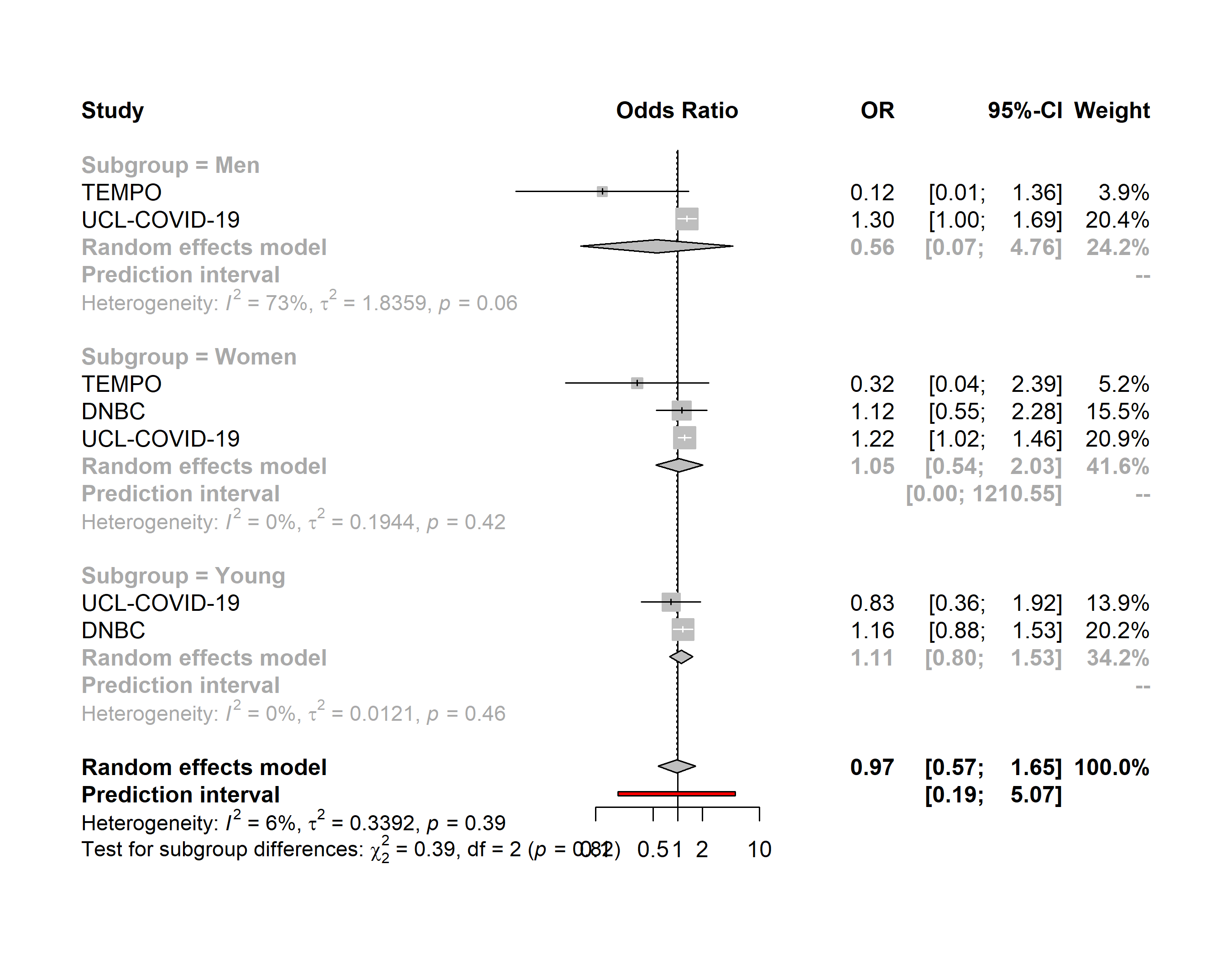


**High life satisfaction**

Household density ≥43m^2^ vs <43m^2^


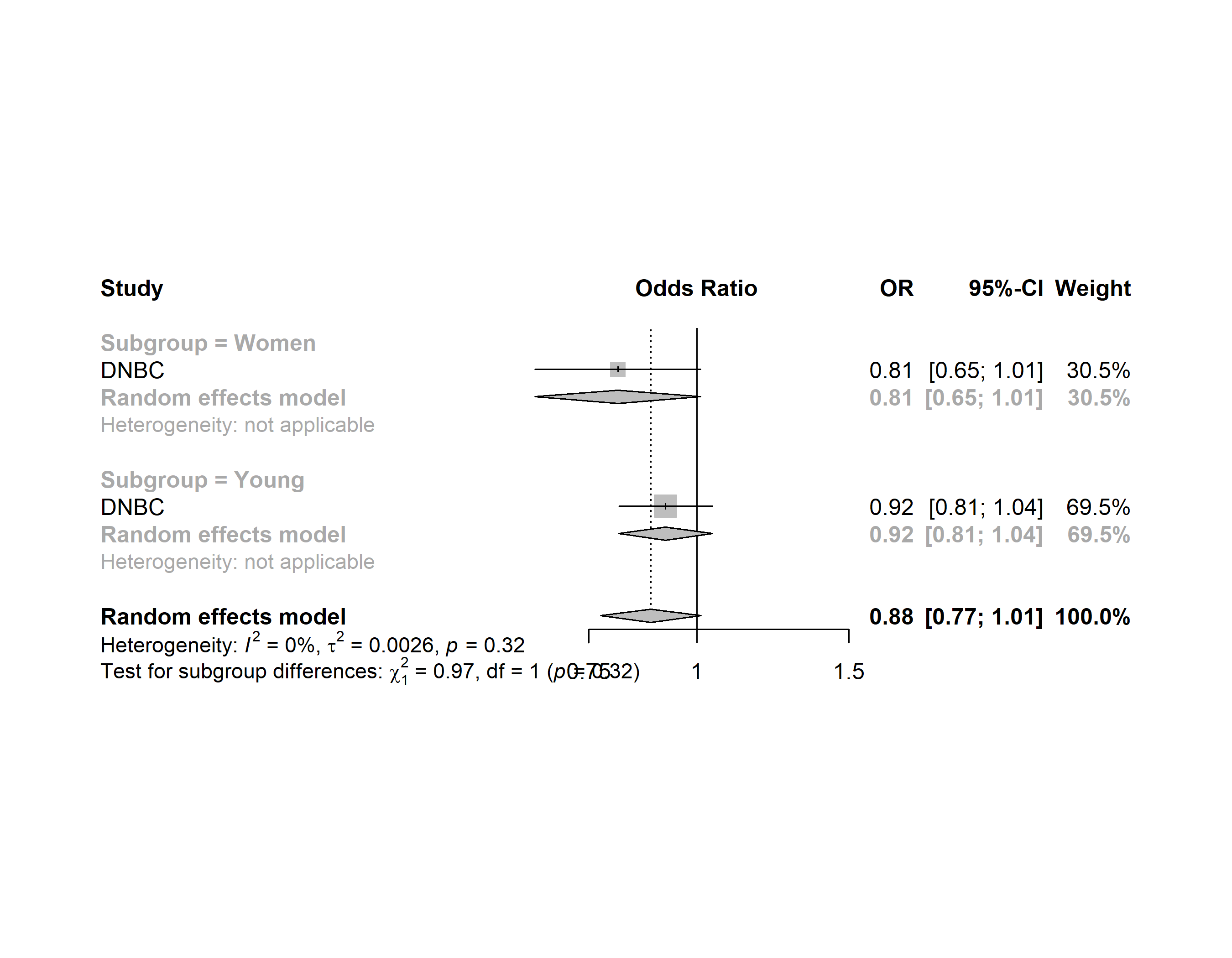


**High life satisfaction**

Household crowded vs. ideal


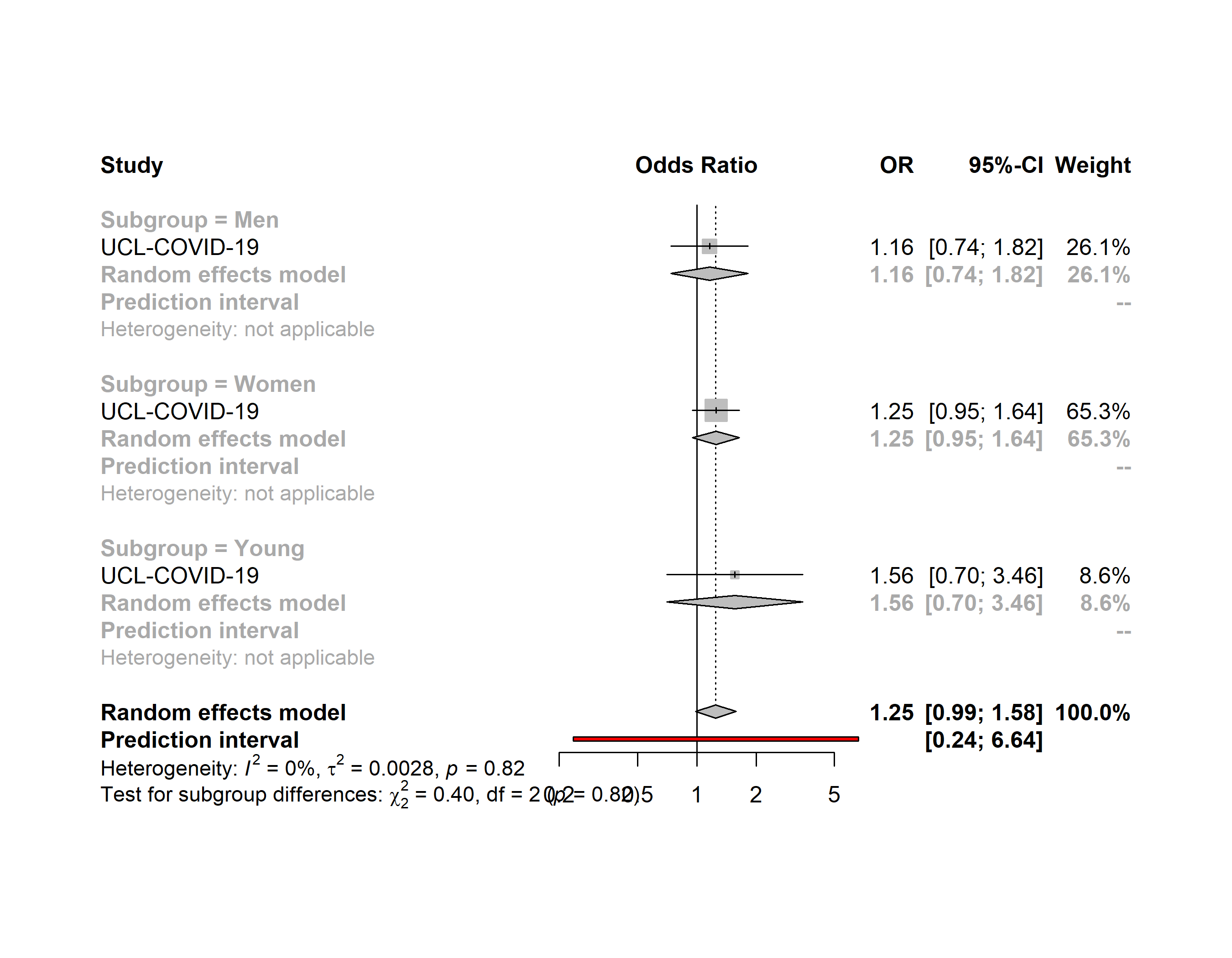


**High life satisfaction**

Household underoccupied vs. ideal


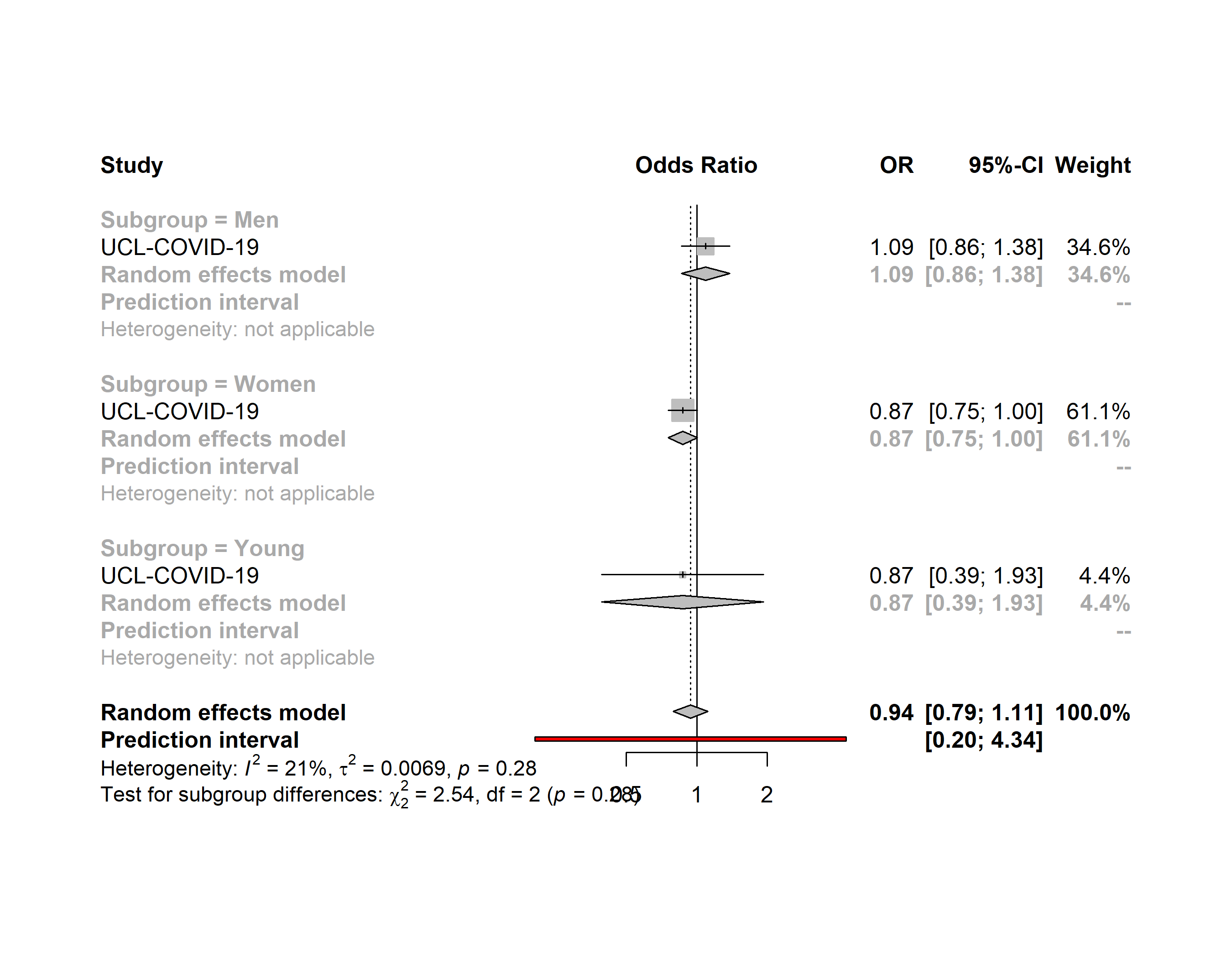


**High life satisfaction**

Households with children vs. Adults-only households


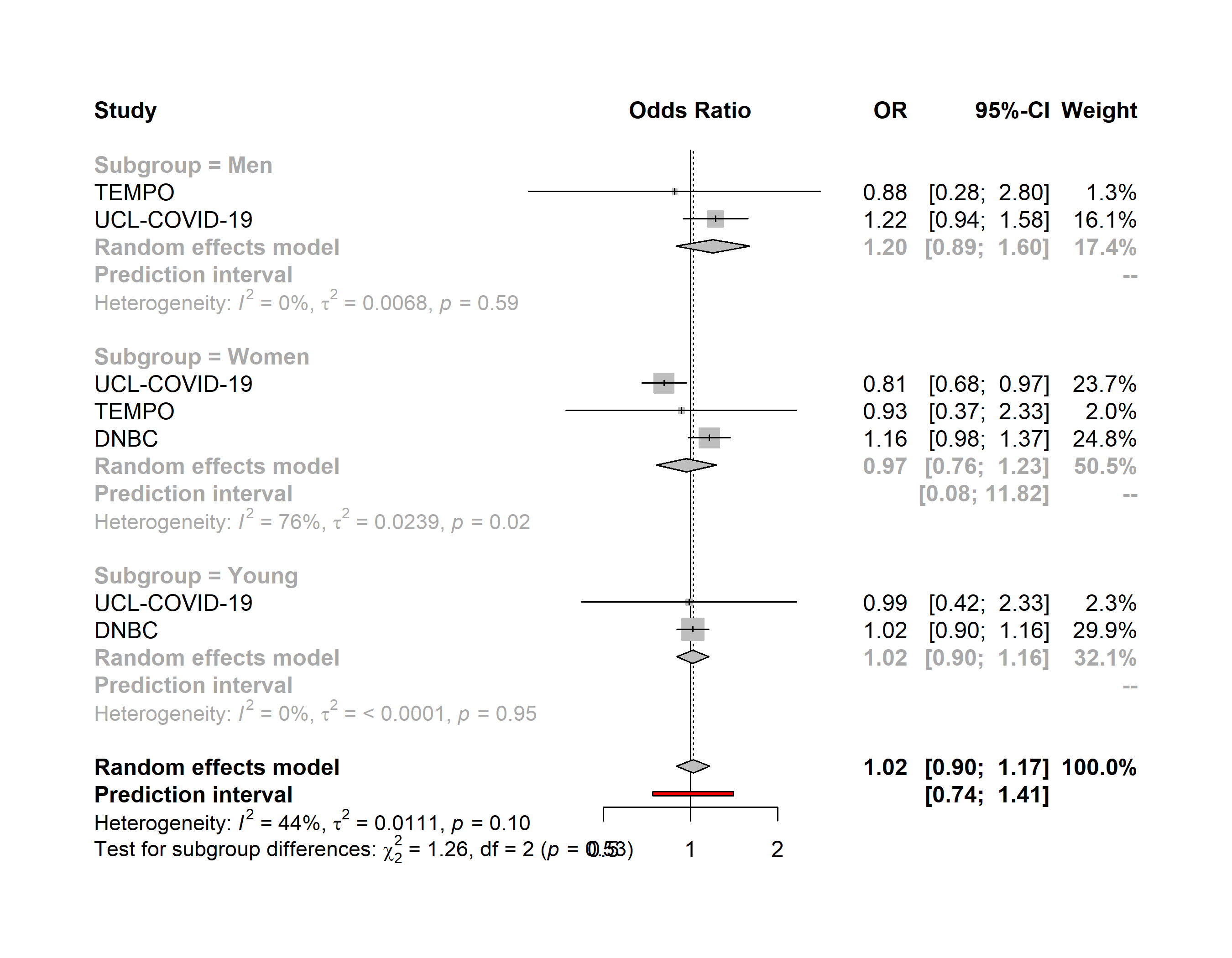


**High life satisfaction**

Living alone vs. Adults-only households


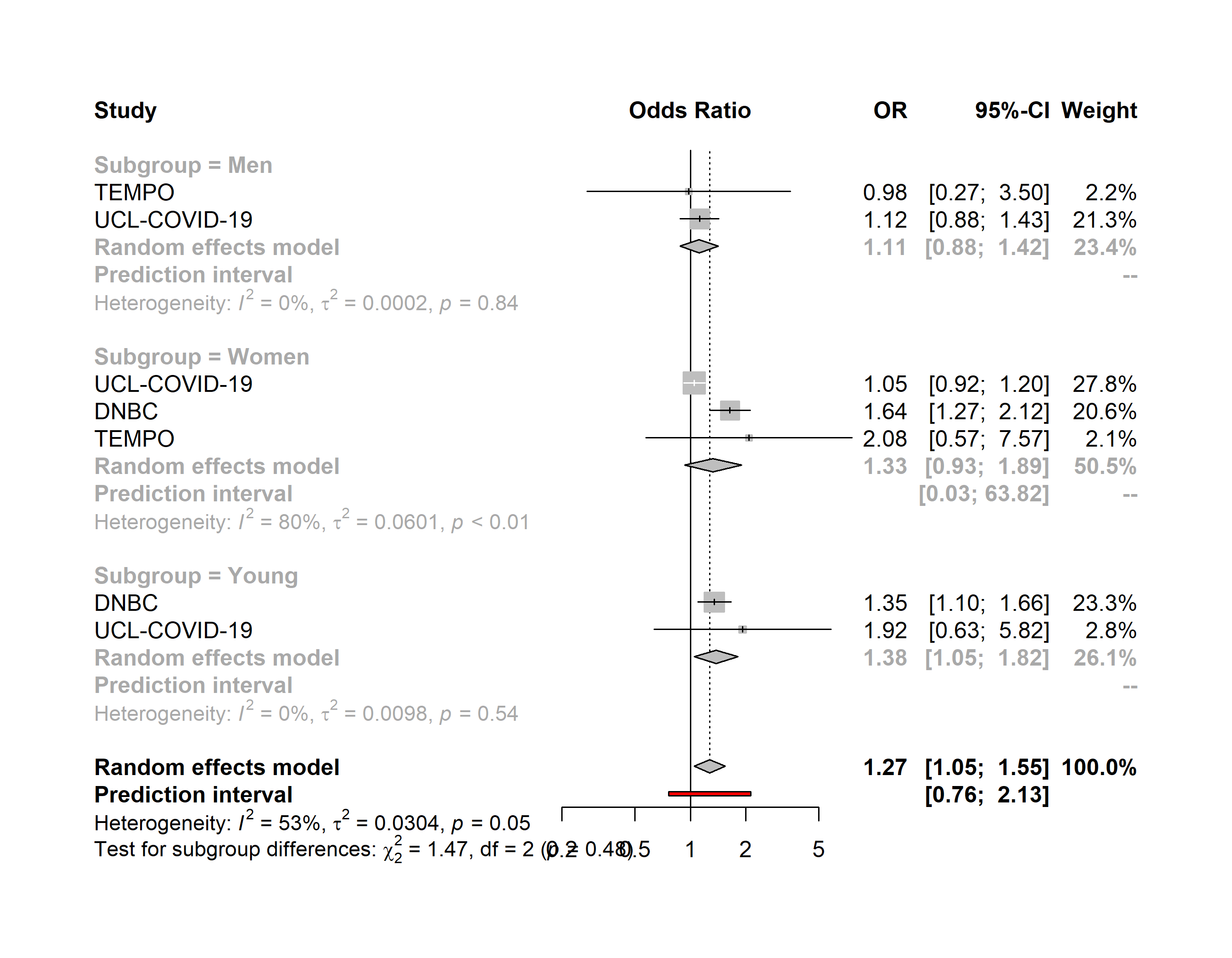


**High life satisfaction**

Apartment vs. House


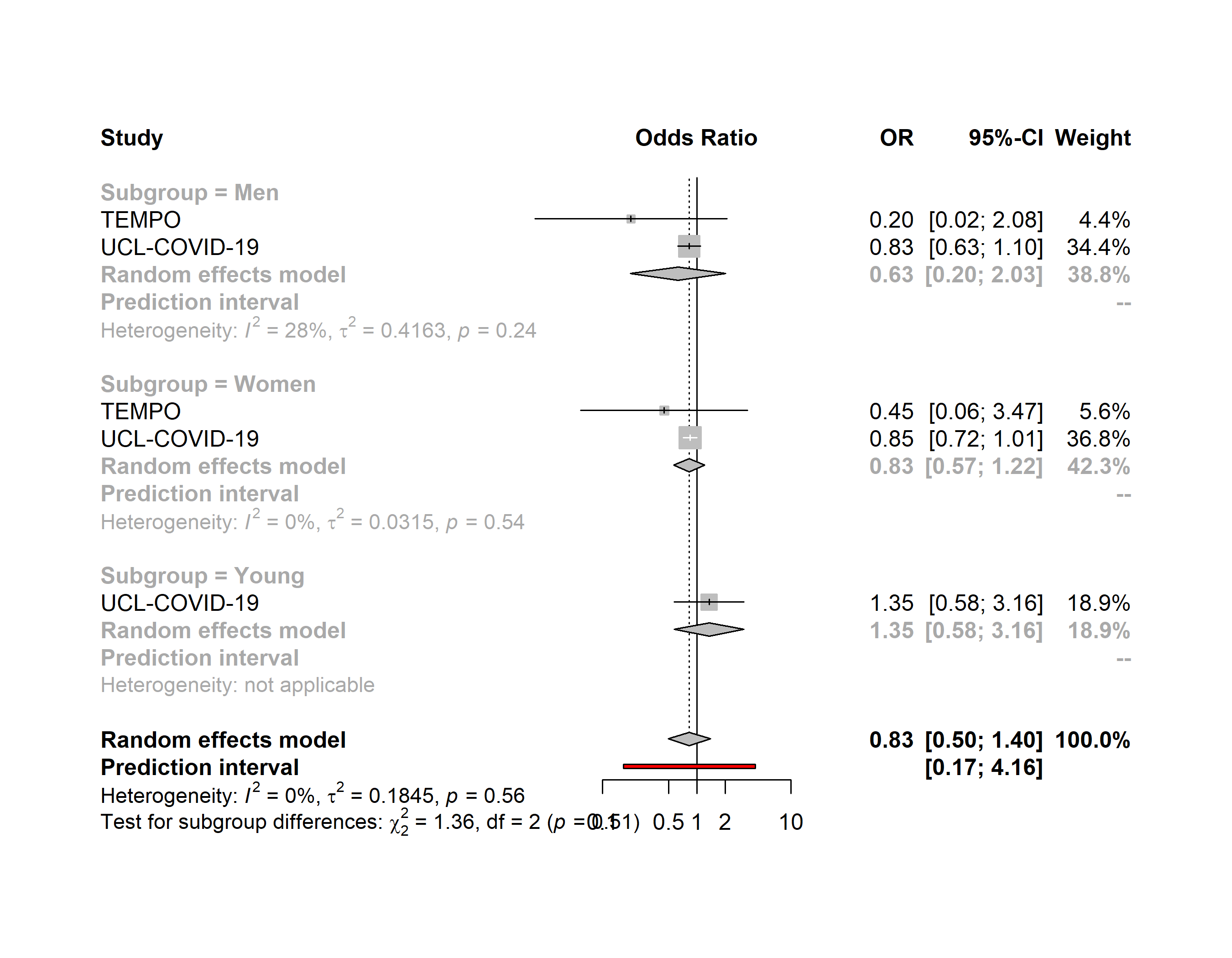


**High life satisfaction**

Semi-urban environment vs. Urban environment


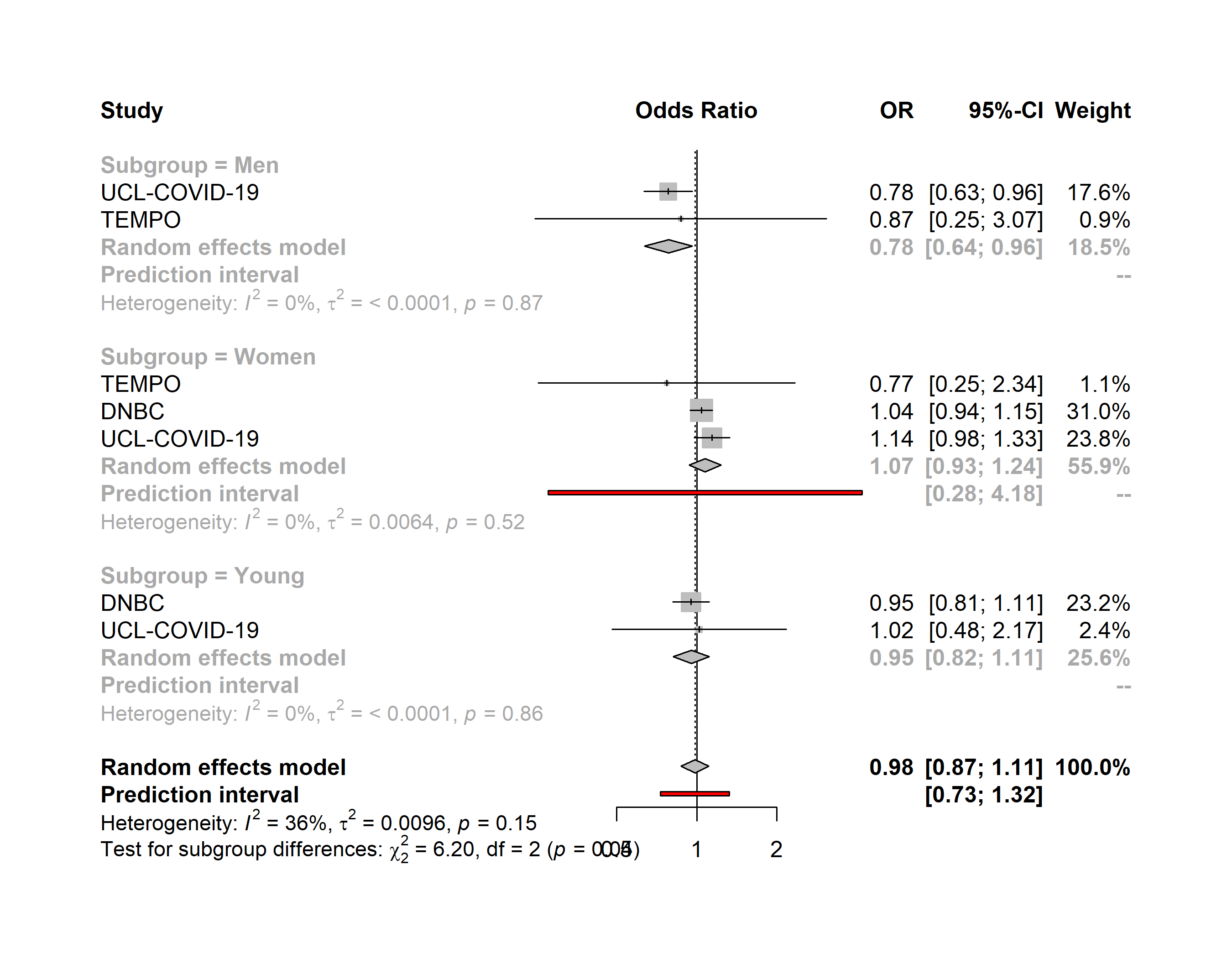


**High life satisfaction**

Rural environment vs. Urban environment


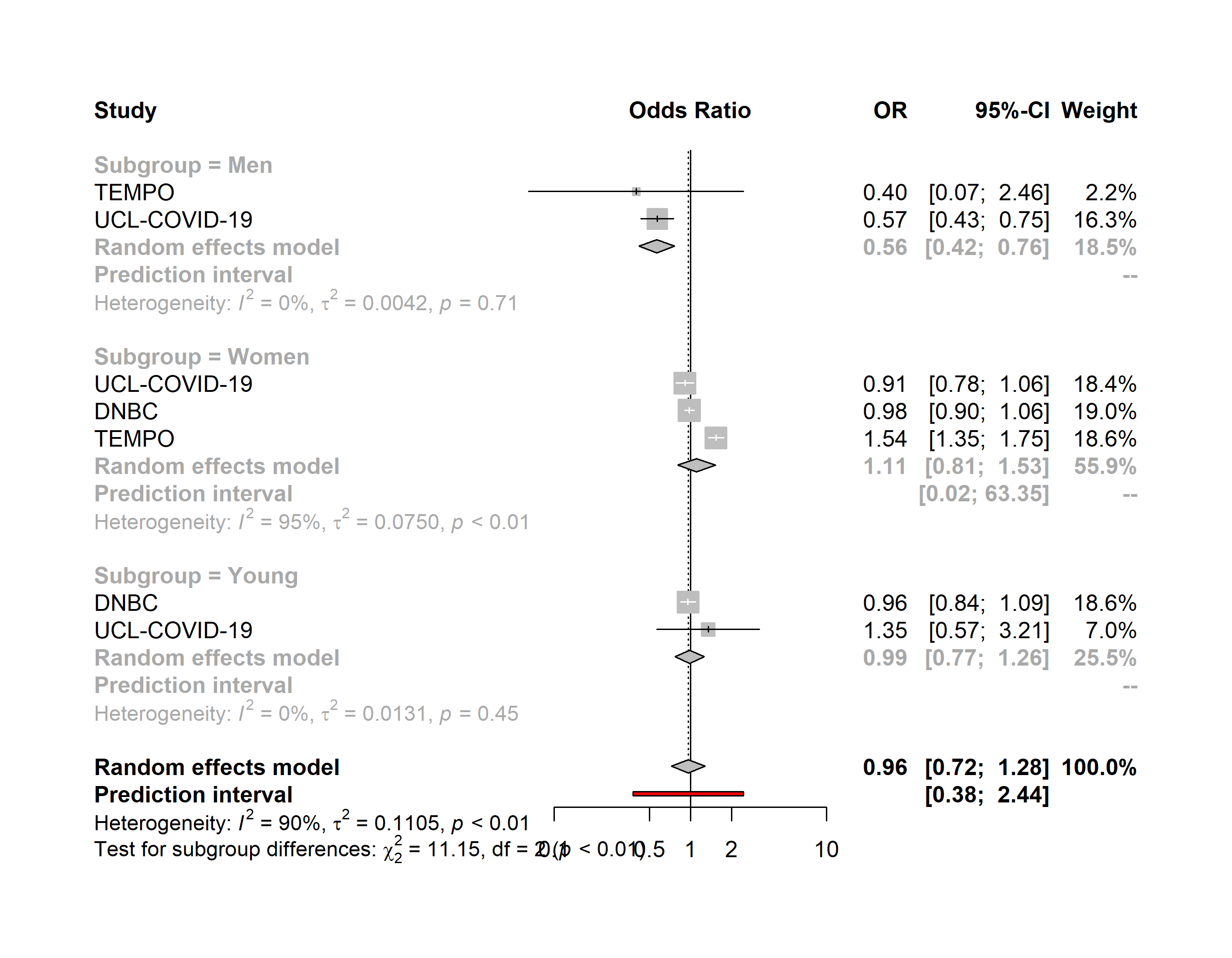

Supplement: Supplementary file 2 — Supplementary Information 2. [file 41598_2022_9316_MOESM2_ESM.docx]
