# Supplementary material for: Housing environment and mental health of Europeans during the COVID-19 pandemic: a cross-country comparison
Source: Sci Rep. 2022 Apr 4;12:5612. doi: 10.1038/s41598-022-09316-4 (PMC8978496; doi:10.1038/s41598-022-09316-4)
Supplement: Supplementary file 4 — Supplementary Table S1. [file 41598_2022_9316_MOESM4_ESM.pdf]

Supplemental Table 1. Descriptive statistics of the four cohorts (N=69,136).

**Variables**		**DNBC**		**TEMPO**		**Constances*,****		**UCL Covid-19 Social Study***		
		Young people	Women	Men	Women	Men	Women	Young people	Men	Women
**N**		9,205	12,684	140	284	13,919	14,252	499	4,561	13,592
**Age**	Mean (SD)	20 (1)	50 (4)	40 (3)	40 (4)	52.3% (51.9;52.7)	50.4% (50.0;50.7)	22 (2.26)	55 (15)	49 (15)
	Median (Q1, Q3)	20 (19;21)	50 (47;53)	41 (38; 43)	41 (38; 42)	51.4% (50.8;52.1)	49.2% (48.6;49.8)	22 (20; 24)	56 (42; 68)	48 (36; 61)
**Age categories**	<20	5,362 (58.3%)	-	-	-	-	-	23.2%	-	-
	20-25	3,843 (41.7%)	-	-	-	-	-	76.8%	-	-
	26-34	-	-	10 (7.1%)	26 (9.2%)	1,079 (12.3%)	1,393 (16.3%)	-	13.7%	21.9%
	35-44	-	779 (6.8%)	118 (84.3%)	241 (84.9%)	2,553 (20.4%)	2,970 (21.03%)	-	15.8%	20.8%
	45-54	-	8,670 (75.9%)	12 (8.6%)	17 (6.0%)	2,884 (21.9%)	3,274 (22.0%)	-	18.4%	21.7%
	55-64	-	1,964 (17.2%)	-	-	3,306 (18.9%)	3,442 (20.5%)	-	18.3%	16.5%
	65+	-	3 (0.0%)	-	-	4,097 (26.2%)	3,173 (20.0%)	-	33.8%	19.1%
**Sex**	Female	6,333 (68.8%)	12,684 (100%)	-	284 (100%)	-	100%	60.2%	-	100%
	Male	2,872 (31.2%)	-	140 (100%)	-	100%	-	39.8%	100%	-
**Educational attainment**	Lower secondary	4,647 (50.5%)	179 (1.4%)	5 (3.6%)	4 (1.4%)	7.3% (6.5;8.2)	6.5% (5.8;7.3)	17.3%	37.5%	32.2%
	Upper secondary	4,445 (48.3%)	4,051 (31.9%)	14 (10.0%)	21 (7.5%)	39.5% (38.1;40.9)	29.8% (28.6;30.9)	54.4%	31.3%	30.5%
	Tertiary	39 (0.4%)	8,408 (66.3%)	121 (86.4%)	257 (91.1%)	53.0% (51.6;54.4)	63.6% (62.3;64.8)	28.3%	31.2%	37.2%
	Other	74 (0.8%)	46 (0.4%)	-	-	-	-	-	-	-
**NCD diagnosis**	No	7,862 (85.4%)	8,907 (70.2%)	64 (45.7%)	127 (44.7%)	56.1% (54.7;57.4)	58.5% (57.3;59.6)	82.7%	52.2%	58.5%
	Yes	1,343 (14.6%)	3,777 (29.8%)	76 (54.3%)	157 (55.3%)	43.8% (42.5;45.2)	41.4% (40.3;42.6)	17.3%	47.8%	41.5%
**Psychiatric diagnosis at any**	No	7,495 (81.4%)	11,438 (90.2%)	126 (90.0%)	244 (85.9%)	95.7% (95.2;96.3)	96.1% (95.6;96.5)	72.4%	85.7%	78.4%
	Yes	1,710 (18.6%)	1,246 (9.8%)	14 (10.0%)	40 (14.1%)	4.2% (3.6;4.7)	3.8% (3.4;4.3)	27.6%	14.3%	21.7%
**Direct access to outdoor**	No	325 (3.5%)	98 (0.8%)	87 (62.1%)	193 (68.0%)	8.4% (7.7;9.1)	7.9% (7.2;8.5)	25.6%	14.4%	13.4%
	Yes	8,880 (96.5%)	12,586(99.2%)	53 (37.9%)	91 (32.0%)	91.5% (90.8;92.2)	92.0% (91.4;92.7)	74.4%	85.6%	86.6%
**Household density**	<43 m2	6,198 (67.3%)	6,922 (54.6%)	-	-	28.3% (27.0;29.5)	28.2% (27.1;29.3)	-	-	-
	≥43 m2	3,007 (32.7%)	5,762 (45.4%)	-	-	71.7% (70.5;73.0)	71.8% (70.6;73.1)	-	-	-
**Household crowing**	Ideal	-	-	-	-	35.5% (34.2;36.8)	36.8% (35.7;38.0)	28.7%	24.1%	22.4%
	Crowded	-	-	-	-	5.0% (4.3;5.6)	4.9% (4.4;5.5)	28.5%	6.6%	6.9%
	Under-occupied	-	-	-	-	59.4% (58.1;60.7)	58.1% (56.9;59.3)	42.8%	69.3%	70.7%
**Household composition**	Single	459 (10.9%)	337 (10.8%)	26 (18.6%)	19 (6.7%)	15.2% (14.2;16.1)	16.2% (15.3;17.1)	8.3%	20.6%	19.0%
	With children	1,426 (33.9%)	1,500 (47.9%)	89 (63.6%)	227 (79.9%)	30.2% (29.0;31.4)	33.5% (32.4;34.7)	23.3%	19.1%	29.4%
	Adults only	2,325 (55.2%)	1,296 (41.4%)	25 (17.9%)	38 (13.4%)	54.5% (53.1;55.8)	50.1% (48.9;51.3)	68.4%	60.3%	51.6%
**Urbanicity**	Urban	3,229 (35.1%)	3,808 (30.0%)	100 (71.4%)	218 (76.8%)	79.5% (78.4;80.7)	77.7% (76.7;78.7)	53.4%	32.3%	32.4%
	Semi-urban	2,045 (22.2%)	3,186 (25.1%)	24 (17.1%)	31 (10.9%)	-	-	33.8%	45.0%	46.9%
	Rural	3,931 (42.7%)	5,686 (44.8%)	16 (11.4%)	35 (12.3%)	20.4% (19.2;21.5)	22.2% (21.2;23.2)	12.7%	22.7%	20.6%
**Loneliness**	Not lonely	2,707 (29.4%)	7,075 (55.8%)	83 (59.3%)	167 (58.8%)	3.9% (3.3;4.5)	4.2% (3.7;4.7)	32.5%	58.4%	48.4%
	Moderately lonely	4,255 (46.2%)	4,801 (37.9%)	46 (32.9%)	99 (34.9%)	6.6% (5.9;7.3)	9.8% (9.1;10.5)	37.9%	25.8%	31.5%
	Very lonely	2,243 (24.4%)	808 (6.4%)	11 (7.9%)	18 (6.3%)	89.3% (88.5;90.2)	85.8% (85.0;86.7)	29.6%	15.8%	20.1%
**Life satisfaction**	Low	3,402 (37.0%)	-	-	-	-	-	46.7%	-	-
	Medium	4,759 (51.7%)	-	-	-	-	-	45.9%	-	-
	High	1,044 (11.3%)	-	-	-	-	-	7.4%	-	-
	Suffering	-	1,691 (13.3%)	4 (2.9%)	7 (2.5%)	-	-	-	21.4%	25.2%
	Struggling	-	3,098 (24.4%)	29 (20.7%)	50 (17.6%)	-	-	-	25.4%	28.9%
	Thriving	-	7,895 (62.2%)	107 (76.4%)	227 (79.9%)	-	-	-	53.2%	46.0%
**Anxiety**	No/mild	4,329 (47.0%)	7,317 (57.7%)	-	-	94.6% (93.9;95.2)	90.6% (89.9;91.3)	72.0%	87.1%	77.3%
	Moderate	2,646 (28.7%)	3,585 (28.3%)	-	-	3.5% (3.0;4.0)	6.3% (5.7;6.9)	11.8%	7.6%	13.3%
	Severe	2,230 (24.2%)	1,782 (14.0%)	-	-	1.8% (1.4;2.2)	2.9% (2.5;3.3)	16.1%	5.4%	9.4%

*For Constances and the UCL COVID-19 Social Study, only percentages are reported, as due to the weighting strategy, the effective sample sizes do not equate to the actual sample sizes.

**For Constances, percentages are reported with 95% confidence intervals.
