# Supplementary material for: Housing environment and mental health of Europeans during the COVID-19 pandemic: a cross-country comparison
Source: Sci Rep. 2022 Apr 4;12:5612. doi: 10.1038/s41598-022-09316-4 (PMC8978496; doi:10.1038/s41598-022-09316-4)
Supplement: Supplementary file 5 — Supplementary Table S2. [file 41598_2022_9316_MOESM5_ESM.pdf]

Supplemental Table 2. Odds ratios for reporting severe loneliness / severe anxiety / low life satisfaction compared to the reference values in the four cohorts (N=69,136) using four different models. Unadjusted model.

LONELINESS

Exposure		DNBC		TEMPO		Constances		UCL Covid-19 Social Study		
		Young people	Women	Men	Women	Men	Women	Young people	Men	Women
**Access to outdoor facilities**	Yes	Ref	Ref	Ref	Ref	Ref	Ref	Ref	Ref	Ref
	No	1.16 [0.94,1.42]	3.41 [2.03,5.71]	0.5 [0.25, 0.99]	1.31 [0.79, 2.19]	2.34 [1.98,2.75]	1.84 [1.58,2.14]	2.48 [1.15,5.36]	1.26 [0.97,1.63]	1.21 [1.02,1.44]
**Household density**	<43 m2	Ref	Ref	N/A	N/A	Ref	Ref	N/A	N/A	N/A
	≥43 m2	0.95 [0.89,1.03]	1.23 [1.15,1.32]	N/A	N/A	0.90 [0.79,1.03]	1.02 [0.92,1.14]	N/A	N/A	N/A
**Household crowding**	Ideal	N/A	N/A	N/A	N/A	Ref	Ref	Ref	Ref	Ref
	Crowded	N/A	N/A	N/A	N/A	1.43 [1.12,1.83]	1.15 [0.92,1.44]	0.63 [0.32,1.24]	1.32 [0.89,1.94]	1.44 [1.10,1.87]
	Underoccupied	N/A	N/A	N/A	N/A	0.42 [0.37,0.47]	0.60 [0.54,0.66]	1.08 [0.57,2.03]	0.62 [0.50,0.78]	0.67 [0.59,0.77]
**Household composition**	Adults-only households	Ref	Ref	Ref	Ref	Ref	Ref	Ref	Ref	Ref
	Households with children	1.00 [0.89,1.12]	0.84 [0.72,0.97]	0.74 [0.31, 1.83]	1.3 [0.65, 2.72]	1.20 [1.01,1.41]	1.27 [0.99,1.61]	2.16 [1.11,4.18]	1.27 [1.00,1.59]	1.31 [1.16,1.48]
	Single households (living alone)	1.63 [1.32,2.00]	2.62 [2.07,3.31]	1 [0.34, 2.95]	3.76 [1.21, 11.96]	10.19 [8.88,11.70]	7.39 [6.04,9.03]	2.35 [0.61,9.04]	3.33 [2.68,4.12]	2.21 [1.92,2.53]
**Dwelling type**	House	N/A	N/A	Ref	Ref	Ref	Ref	Ref	Ref	Ref
	Apartment	N/A	N/A	1.68 [0.84, 3.33]	0.67 [0.39, 1.12]	2.38 [2.11,2.67]	1.79 [1.63,1.98]	0.95 [0.45,2.00]	0.90 [0.70,1.15]	0.99 [0.85,1.16]
**Urbanicity**	Urban	Ref	Ref	Ref	Ref	Ref	Ref	Ref	Ref	Ref
	Semi-urban	0.98 [0.89,1.08]	1.07 [0.98,1.17]	0.37 [0.13, 0.97]	0.96 [0.45, 1.98]	N/A	N/A	1.06 [0.62,1.83]	0.92 [0.75,1.13]	1.05 [0.93,1.18]
	Rural	1.05 [0.97,1.14]	1.05 [0.96,1.13]	0.35 [0.09, 1.07]	0.87 [0.41, 1.78]	0.87 [0.74,1.01]	0.84 [0.74,0.95]	2.31 [1.14,4.67]	0.81 [0.63,1.04]	0.89 [0.78,1.03]

ANXIETY

Exposure		DNBC		TEMPO		Constances		UCL Covid-19 Social Study		
		Young people	Women	Men	Women	Men	Women	Young people	Men	Women
**Access to outdoor facilities**	Yes	Ref	Ref	N/A	N/A	Ref	Ref	Ref	Ref	Ref
	No	1.32 [1.08,1.61]	1.19 [0.81,1.74]	N/A	N/A	1.80 [1.41, 2.29]	1.20 [0.98, 1.47]	1.48 [0.70,3.14]	1.28 [0.89,1.83]	1.22 [1.00,1.49]
**Household density**	<43 m2	Ref	Ref	N/A	N/A	Ref	Ref	N/A	N/A	N/A
	≥43 m2	0.89 [0.83,0.96]	0.98 [0.91,1.05]	N/A	N/A	1.05 [0.74,1.49]	0.71 [0.63,0.81]	N/A	N/A	N/A
**Household crowding**	Ideal	N/A	N/A	N/A	N/A	Ref	Ref	Ref	Ref	Ref
	Crowded	N/A	N/A	N/A	N/A	1.37 [0.94,1.98]	1.22 [0.93,1.61]	1.51 [0.71,3.19]	1.38 [0.81,2.36]	1.30 [0.97,1.74]
	Underoccupied	N/A	N/A	N/A	N/A	0.61 [0.52,0.73]	0.76 [0.67,0.86]	1.31 [0.63,2.72]	0.48 [0.34,0.68]	0.59 [0.50,0.69]
**Household composition**	Adults-only households	Ref	Ref	N/A	N/A	Ref	Ref	Ref	Ref	Ref
	Households with children	0.95 [0.85,1.06]	1.04 [0.90,1.20]	N/A	N/A	1.41 [1.17,1.70]	1.10 [0.88,1.38]	0.72 [0.35,1.47]	1.19 [0.83,1.69]	1.38 [1.19,1.60]
	Single households (living alone)	1.20 [1.00,1.45]	1.13 [0.90,1.43]	N/A	N/A	2.02 [1.63,2.51]	1.08 [0.81,1.43]	2.27 [0.65,7.90]	0.96 [0.69,1.34]	0.80 [0.68,0.94]
**Dwelling type**	House	N/A	N/A	N/A	N/A	Ref	Ref	Ref	Ref	Ref
	Apartment	N/A	N/A	N/A	N/A	1.63 [1.38,1.93]	1.02 [0.90,1.16]	0.58 [0.26,1.30]	0.89 [0.61,1.31]	1.05 [0.86,1.27]
	Urban	Ref	Ref	N/A	N/A	Ref	Ref	Ref	Ref	Ref

**Urbanicity**	Semi-urban	0.88 [0.80,0.97]	1.03 [0.94,1.13]	N/A	N/A	N/A	N/A	0.84 [0.44,1.62]	0.99 [0.70,1.40]	1.05 [0.90,1.22]
	Rural	0.93 [0.84,1.02]	1.03 [0.93,1.15]	N/A	N/A	0.75 [0.59,0.94]	1.05 [0.91,1.22]	1.28 [0.57,2.88]	0.91 [0.59,1.39]	0.88 [0.73,1.06]

LIFE SATISFACTION

**Exposure**		**DNBC**		**TEMPO**		**Constances**		**UCL Covid-19 Social Study**		
		**Young people**	**Women**	**Men**	**Women**	**Men**	**Women**	**Young people**	**Men**	**Women**
**Access to outdoor facilities**	Yes	Ref	Ref	Ref	Ref	N/A	N/A	Ref	Ref	Ref
	No	1.1 [0.89, 1.35]	1.35 [0.92, 2.00]	0.48 [0.22, 1.06]	0.68 [0.37, 1.25]	N/A	N/A	1.18 [0.56, 2.5]	1.35 [1.06, 1.72]	1.33 [1.12, 1.56]
**Household density**	<43 m2	Ref	Ref	N/A	N/A	N/A	N/A	N/A	N/A	N/A
	≥43 m2	0.93 [0.85, 1.00]	1.01 [0.91, 1.11]	N/A	N/A	N/A	N/A	N/A	N/A	N/A
**Household crowding**	Ideal	N/A	N/A	N/A	N/A	N/A	N/A	Ref	Ref	Ref
	Crowded	N/A	N/A	N/A	N/A	N/A	N/A	1.12 [0.56, 2.27]	1.64 [1.02, 2.63]	1.43 [1.10, 1.89]
	Underoccupied	N/A	N/A	N/A	N/A	N/A	N/A	0.93 [0.45, 1.92]	0.75 [0.61, 0.83]	0.67 [0.58, 0.76]
**Household composition**	Adults-only households	Ref	Ref	Ref	Ref	N/A	N/A	Ref	Ref	Ref
	Households with children	1.03 [0.92, 1.15]	1.08 [0.93, 1.23]	0.69 [0.26, 2.00]	0.85 [0.38, 2.08]	N/A	N/A	1.23 [0.58, 2.56]	1.41 [1.12, 1.75]	1.14 [1.01, 1.28]
	Single households (living alone)	1.28 [1.06, 1.56]	1.75 [1.39, 2.22]	1.19 [0.36, 4.00]	2.17 [0.65, 7.14]	N/A	N/A	2.44 [0.67, 9.09]	1.79 [1.45, 2.22]	1.23 [1.09, 1.39]
**Dwelling type**	House	N/A	N/A	Ref	Ref	N/A	N/A	Ref	Ref	Ref
	Apartment	N/A	N/A	1.72 [0.78, 3.85]	1.37 [0.72, 2.5]	N/A	N/A	1.2 [0.55, 2.63]	0.83 [0.65, 1.08]	0.93 [0.79, 1.08]
**Urbanicity**	Urban	Ref	Ref	Ref	Ref	N/A	N/A	Ref	Ref	Ref
	Semi-urban	1.11 [0.91, 1.12]	1.04 [0.94, 1.14]	0.77 [0.24, 2.13]	0.76 [0.25, 1.96]	N/A	N/A	1.12 [0.60, 2.08]	0.79 [0.65, 0.95]	1.16 [1.01, 1.33]
	Rural	1.06 [0.98, 1.16]	0.98 [0.90, 1.06]	0.4 [0.06, 1.56]	1.39 [0.58, 3.13]	N/A	N/A	1.85 [0.85, 4.00]	0.58 [0.46, 0.74]	0.85 [0.75, 0.98]
