# Supplementary material for: Housing environment and mental health of Europeans during the COVID-19 pandemic: a cross-country comparison
Source: Sci Rep. 2022 Apr 4;12:5612. doi: 10.1038/s41598-022-09316-4 (PMC8978496; doi:10.1038/s41598-022-09316-4)
Supplement: Supplementary file 6 — Supplementary Table S3. [file 41598_2022_9316_MOESM6_ESM.pdf]

Supplemental Table 3. Odds ratio of reporting severe loneliness / severe anxiety / low life satisfaction compared to the reference values in the four cohorts (N=69,136). Model 1.

LONELINESS

Exposure		DNBC		TEMPO		Constances		UCL Covid-19 Social Study		
		**Young people**	**Women**	**Men**	**Women**	**Men**	**Women**	**Young people**	**Men**	**Women**
**Access to outdoor facilities**	Yes	Ref	Ref	Ref	Ref	Ref	Ref	Ref	Ref	Ref
	No	1.33 [1.08,1.64]	3.23 [1.84,5.67]	0.52 [0.26, 1.05]	1.44 [0.86, 2.45]	2.13 [1.80,2.52]	1.87 [1.61,2.17]	2.54 [1.19,5.44]	1.29 [0.99,1.67]	1.13 [0.95,1.35]
**Household density**	<43 m2	Ref	Ref	N/A	N/A	Ref	Ref	N/A	N/A	N/A
	≥43 m2	0.96 [0.89,1.04]	1.25 [1.16,1.35]	N/A	N/A	1.08 [0.94,1.25]	1.00 [0.88,1.12]	N/A	N/A	N/A
**Household crowding**	Ideal	N/A	N/A	N/A	N/A	Ref	Ref	Ref	Ref	Ref
	Crowded	N/A	N/A	N/A	N/A	1.40 [1.09,1.79]	1.20 [0.96,1.50]	0.58 [0.29,1.16]	1.13 [0.77,1.64]	1.25 [0.96,1.62]
	Underoccupied	N/A	N/A	N/A	N/A	0.44 [0.38,0.50]	0.57 [0.52,0.64]	1.00 [0.54,1.88]	0.75 [0.59,0.94]	0.79 [0.69,0.90]
**Household composition**	Adults-only households	Ref	Ref	Ref	Ref	Ref	Ref	Ref	Ref	Ref
	Households with children	0.92 [0.82,1.03]	0.84 [0.71,0.99]	0.67 [0.27, 1.68]	1.61 [0.77, 3.56]	1.00 [0.84,1.20]	1.17 [0.91,1.49]	1.98 [0.96,4.09]	0.92 [0.70,1.19]	1.01 [0.88,1.15]
	Single households (living alone)	1.78 [1.47,2.16]	2.53 [1.97,3.24]	0.89 [0.3, 2.67]	4 [1.26, 13.07]	9.40 [8.16,10.83]	7.38 [6.04,9.02]	2.70 [0.91,8.01]	3.50 [2.81,4.36]	2.58 [2.23,2.98]
**Dwelling type**	House	N/A	N/A	Ref	Ref	Ref	Ref	Ref	Ref	Ref
	Apartment	N/A	N/A	1.63 [0.8, 3.32]	0.63 [0.36, 1.07]	2.27 [2.02,2.57]	1.83 [1.66,2.01]	0.70 [0.31,1.54]	0.87 [0.68,1.13]	0.94 [0.80,1.11]
**Urbanicity**	Urban	Ref	Ref	Ref	Ref	Ref	Ref	Ref	Ref	Ref
	Semi-urban	0.92 [0.83,1.01]	1.05 [0.95,1.16]	0.37 [0.12, 0.95]	0.85 [0.39, 1.8]	N/A	N/A	1.20 [0.67,2.14]	1.07 [0.88,1.31]	1.03 [0.91,1.16]
	Rural	0.97 [0.90,1.06]	1.01 [0.93,1.10]	0.36 [0.09, 1.15]	0.83 [0.38, 1.75]	0.88 [0.75,1.04]	0.84 [0.74,0.95]	2.70 [1.27,5.74]	1.01 [0.77,1.31]	0.91 [0.79,1.06]

ANXIETY

Exposure		DNBC		TEMPO		Constances		UCL Covid-19 Social Study		
		**Young people**	**Women**	**Men**	**Women**	**Men**	**Women**	**Young people**	**Men**	**Women**
**Access to outdoor facilities**	Yes	Ref	Ref	N/A	N/A	Ref	Ref	Ref	Ref	Ref
	No	1.39 [1.13,1.71]	1.20 [0.80,1.80]	N/A	N/A	1.55 [1.21, 1.99]	1.12 [0.91,1.38]	1.48 [0.71,3.08]	1.33 [0.92,1.92]	1.14 [0.93,1.39]
**Household density**	<43 m2	Ref	Ref	N/A	N/A	Ref	Ref	N/A	N/A	N/A
	≥43 m2	0.88 [0.81,0.95]	0.95 [0.88,1.02]	N/A	N/A	0.93 [0.77, 1.13]	0.85 [0.74, 0.97]	N/A	N/A	N/A
**Household crowding**	Ideal	N/A	N/A	N/A	N/A	Ref	Ref	Ref	Ref	Ref
	Crowded	N/A	N/A	N/A	N/A	1.26 [0.87,1.82]	1.13 [0.85,1.49]	1.64 [0.76,3.52]	1.19 [0.66,2.16]	1.07 [0.82,1.42]
	Underoccupied	N/A	N/A	N/A	N/A	0.72 [0.60 ,0.86]	0.83 [0.74,0.95]	1.69 [0.79,3.58]	0.61 [0.44,0.85]	0.71 [0.60,0.85]
**Household composition**	Adults-only households	Ref	Ref	N/A	N/A	Ref	Ref	Ref	Ref	Ref
	Households with children	0.96 [0.85,1.08]	1.04 [0.89,1.22]	N/A	N/A	1.06 [0.86,1.30]	1.12 [0.97,1.30]	0.53 [0.25,1.13]	0.83 [0.57,1.19]	1.01 [0.85,1.19]
	Single households (living alone)	1.39 [1.14,1.68]	1.16 [0.91,1.48]	N/A	N/A	1.74 [1.39,2.17]	0.96 [0.80,1.14]	3.19 [0.92,11.13]	0.90 [0.63,1.27]	0.93 [0.78,1.11]
**Dwelling type**	House	N/A	N/A	N/A	N/A	Ref	Ref	Ref	Ref	Ref
	Apartment	N/A	N/A	N/A	N/A	1.46 [1.23,1.74]	0.97 [0.85,1.10]	0.49 [0.22,1.09]	0.85 [0.56,1.28]	1.01 [0.83,1.22]
**Urbanicity**	Urban	Ref	Ref	N/A	N/A	Ref	Ref	Ref	Ref	Ref
	Semi-urban	0.85 [0.77,0.93]	1.05 [0.96,1.16]	N/A	N/A	N/A	N/A	0.69 [0.36,1.34]	1.15 [0.83,1.59]	1.00 [0.85,1.17]

	Rural	0.89 [0.80,0.98]	0.97 [0.89,1.06]	N/A	N/A	0.75 [0.59,0.95]	1.01 [0.87,1.17]	1.14 [0.50,2.56]	1.17 [0.77,1.77]	0.90 [0.74,1.08]

LIFE SATISFACTION

**Exposure**		**DNBC**		**TEMPO**		**Constances**		**UCL Covid-19 Social Study**		
		**Young people**	**Women**	**Men**	**Women**	**Men**	**Women**	**Young people**	**Men**	**Women**
**Access to outdoor facilities**	Yes	Ref	Ref	Ref	Ref	N/A	N/A	Ref	Ref	Ref
	No	1.22 [0.99, 1.52]	1.32 [0.88, 2.00]	0.47 [0.20, 1.05]	0.67 [0.36, 1.23]	N/A	N/A	1.18 [0.54, 2.56]	1.37 [1.08, 1.72]	1.25 [1.06, 1.49]
**Household density**	<43 m2	Ref	Ref	N/A	N/A	N/A	N/A	N/A	N/A	N/A
	≥43 m2	0.91 [0.84, 0.99]	0.97 [0.87, 1.09]	N/A	N/A	N/A	N/A	N/A	N/A	N/A
**Household crowding**	Ideal	N/A	N/A	N/A	N/A	N/A	N/A	Ref	Ref	Ref
	Crowded	N/A	N/A	N/A	N/A	N/A	N/A	1.2 [0.58, 2.50]	1.52 [0.96, 2.38]	1.32 [1.02, 1.69]
	Underoccupied	N/A	N/A	N/A	N/A	N/A	N/A	1.05 [0.51, 2.17]	0.87 [0.70, 1.08]	0.77 [0.67, 0.88]
**Household composition**	Adults-only households	Ref	Ref	Ref	Ref	N/A	N/A	Ref	Ref	Ref
	Households with children	1.02 [0.90, 1.15]	1.15 [0.98, 1.37]	0,65 [0.24, 1.92]	0.79 [0.34, 2.04]	N/A	N/A	1.12 [0.50, 2.50]	1.11 [0.86, 1.41]	0.92 [0.81, 1.04]
	Single households (living alone)	1.52 [1.23, 1.85]	1.67 [1.32, 2.13]	1.18 [0.35, 4.00]	2.13 [0.62, 7.14]	N/A	N/A	2.70 [0.79, 9.09]	1.72 [1.39, 2.13]	1.41 [1.23, 1.59]
**Dwelling type**	House	N/A	N/A	Ref	Ref	N/A	N/A	Ref	Ref	Ref
	Apartment	N/A	N/A	1.75 [0.77, 4.00]	1.39 [0.74, 2.56]	N/A	N/A	1.16 [0.50, 2.70]	0.82 [0.64, 1.05]	0.88 [0.76, 1.03]
**Urbanicity**	Urban	Ref	Ref	Ref	Ref	N/A	N/A	Ref	Ref	Ref
	Semi-urban	0.95 [0.85, 1.06]	1.05 [0.95, 1.16]	0.77 [0.24, 2.17]	0.71 [0.23, 1.85]	N/A	N/A	1.16 [0.57, 2.33]	0.85 [0.69, 1.03]	1.12 [0.98, 1.28]
	Rural	1.00 [0.92, 1.10]	0.98 [0.89, 1.06]	0.36 [0.05, 1.49]	1.33 [0.55, 3.03]	N/A	N/A	1.92 [0.83, 4.55]	0.66 [0.52, 0.84]	0.88 [0.78, 1.01]
