# Supplementary material for: Housing environment and mental health of Europeans during the COVID-19 pandemic: a cross-country comparison
Source: Sci Rep. 2022 Apr 4;12:5612. doi: 10.1038/s41598-022-09316-4 (PMC8978496; doi:10.1038/s41598-022-09316-4)
Supplement: Supplementary file 7 — Supplementary Table S4. [file 41598_2022_9316_MOESM7_ESM.pdf]

Supplemental Table 4. Odds ratio of reporting severe loneliness / severe anxiety / low life satisfaction compared to the reference values in the four cohorts (N=69,136). Model 2.

LONELINESS

Exposure		DNBC		TEMPO		Constances		UCL Covid-19 Social Study		
		Young people	Women	Men	Women	Men	Women	Young people	Men	Women
Access to outdoor facilities	Yes	Ref	Ref	Ref	Ref	Ref	Ref	Ref	Ref	Ref
	No	1.29 [1.05,1.59]	1.99 [1.31,3.02]	0.54 [0.27, 1.09]	1.43 [0.85, 2.44]	2.06 [1.74,2.43]	1.84 [1.58,2.13]	2.78 [1.29,6.00]	1.28 [0.99,1.66]	1.09 [0.91,1.31]
Household density	<43 m2	Ref	Ref	N/A	N/A	Ref	Ref	N/A	N/A	N/A
	≥43 m2	0.97 [0.90,1.05]	1.24 [1.15,1.34]	N/A	N/A	1.08 [0.94,1.25]	1.00 [0.89,1.13]	N/A	N/A	N/A
Household crowding	Ideal	N/A	N/A	N/A	N/A	Ref	Ref	Ref	Ref	Ref
	Crowded	N/A	N/A	N/A	N/A	1.37 [1.07,1.76]	1.18 [0.94,1.48]	0.54 [0.27,1.10]	1.18 [0.81,1.72]	1.27 [0.98,1.63]
	Underoccupied	N/A	N/A	N/A	N/A	0.44 [0.39,0.50]	0.58 [0.52,0.64]	0.90 [0.48,1.69]	0.76 [0.61,0.96]	0.83 [0.72,0.96]
Household composition	Adults-only households	Ref	Ref	Ref	Ref	Ref	Ref	Ref	Ref	Ref
	Households with children	0.93 [0.83,1.05]	0.84 [0.71,0.99]	0.7 [0.28, 1.79]	1.73 [0.82, 3.88]	1.03 [0.86,1.23]	1.17 [0.91,1.50]	2.05 [1.00,4.21]	0.91 [0.69,1.19]	1.05 [0.92,1.20]
	Single households (living alone)	1.72 [1.42,2.09]	2.45 [1.91,3.15]	0.9 [0.3, 2.74]	4.08 [1.27, 13.46]	9.35 [8.11,10.78]	7.34 [6.00,8.97]	2.15 [0.86,5.39]	3.43 [2.75,4.27]	2.67 [2.33,3.06]
Dwelling type	House	N/A	N/A	Ref	Ref	Ref	Ref	Ref	Ref	Ref
	Apartment	N/A	N/A	1.55 [0.76, 3.17]	0.64 [0.37, 1.09]	2.21 [1.96,2.50]	1.82 [1.65,2.00]	0.64 [0.28,1.47]	0.82 [0.63,1.06]	0.94 [0.80,1.12]
Urbanicity	Urban	Ref	Ref	Ref	Ref	Ref	Ref	Ref	Ref	Ref
	Semi-urban	0.92 [0.83,1.01]	1.05 [0.95,1.16]	0.36 [0.12, 0.95]	0.88 [0.4, 1.88]	N/A	N/A	1.02 [0.56,1.84]	1.09 [0.89,1.35]	1.04 [0.92,1.17]
	Rural	0.98 [0.90,1.06]	1.00 [0.92,1.09]	0.37 [0.09, 1.19]	0.88 [0.4, 1.85]	0.89 [0.76,1.05]	0.84 [0.74,0.95]	2.38 [1.12,5.09]	0.99 [0.76,1.30]	0.90 [0.78,1.05]

ANXIETY

Exposure		DNBC		TEMPO		Constances		UCL Covid-19 Social Study		
		Young people	Women	Men	Women	Men	Women	Young people	Men	Women
Access to outdoor facilities	Yes	Ref	Ref	N/A	N/A	Ref	Ref	Ref	Ref	Ref
	No	1.22 [0.99,1.51]	1.10 [0.73,1.66]	N/A	N/A	1.46 [1.14,1.88]	1.09 [0.88,1.34]	1.72 [0.79,3.72]	1.32 [0.89,1.95]	1.11 [0.90,1.37]
Household density	<43 m2	Ref	Ref	N/A	N/A	Ref	Ref	N/A	N/A	N/A
	≥43 m2	0.90 [0.83,0.97]	0.94 [0.87,1.01]	N/A	N/A	0.93 [0.76, 1.13]	0.85 [0.74, 0.97]	N/A	N/A	N/A
Household crowding	Ideal	N/A	N/A	N/A	N/A	Ref	Ref	Ref	Ref	Ref
	Crowded	N/A	N/A	N/A	N/A	1.21 [0.84,1.76]	1.10 [0.83,1.46]	1.55 [0.70,3.44]	1.18 [0.61,2.29]	1.09 [0.82,1.46]
	Underoccupied	N/A	N/A	N/A	N/A	0.75 [0.62,0.89]	0.84 [0.74,0.96]	1.34 [0.64,2.83]	0.59 [0.41,0.86]	0.76 [0.64,0.91]
Household composition	Adults-only households	Ref	Ref	N/A	N/A	Ref	Ref	Ref	Ref	Ref
	Households with children	1.00 [0.89,1.13]	1.05 [0.89,1.23]	N/A	N/A	1.10 [0.89,1.36]	1.14 [0.98,1.32]	0.58 [0.27,1.27]	0.85 [0.58,1.24]	1.09 [0.92,1.29]
	Single households (living alone)	1.19 [0.98,1.45]	1.08 [0.84,1.38]	N/A	N/A	1.69 [1.35,2.11]	0.94 [0.79,1.12]	2.07 [0.72,5.91]	0.75 [0.52,1.08]	0.78 [0.65,0.94]
Dwelling type	House	N/A	N/A	N/A	N/A	Ref	Ref	Ref	Ref	Ref
	Apartment	N/A	N/A	N/A	N/A	1.39 [1.17,1.66]	0.96 [0.84,1.08]	0.40 [0.17,0.94]	0.75 [0.48,1.16]	1.00 [0.82,1.22]
Urbanicity	Urban	Ref	Ref	N/A	N/A	Ref	Ref	Ref	Ref	Ref
	Semi-urban	0.84 [0.76,0.93]	1.06 [0.96,1.17]	N/A	N/A	N/A	N/A	0.55 [0.27,1.10]	1.25 [0.86,1.81]	1.01 [0.87,1.19]

	Rural	0.91 [0.82,1.01]	0.96 [0.88,1.04]	N/A	N/A	0.76 [0.60,0.96]	1.02 [0.88,1.18]	0.99 [0.40,2.46]	1.17 [0.75,1.81]	0.87 [0.72,1.06]

LIFE SATISFACTION

**Exposure**		**DNBC**		**TEMPO**		**Constances**		**UCL Covid-19 Social Study**		
		**Young people**	**Women**	**Men**	**Women**	**Men**	**Women**	**Young people**	**Men**	**Women**
**Access to outdoor facilities**	Yes	Ref	Ref	Ref	Ref	N/A	N/A	Ref	Ref	Ref
	No	1.14 [0.92, 1.41]	1.27 [0.83, 1.89]	0.47 [0.21, 1.06]	0.63 [0.34, 1.18]	N/A	N/A	1.32 [0.61, 2.86]	1.35 [1.06, 1.72]	1.23 [1.04, 1.47]
**Household density**	<43 m2	Ref	Ref	N/A	N/A	N/A	N/A	N/A	N/A	N/A
	≥43 m2	0.93 [0.85, 1.00]	0.96 [0.86, 1.08]	N/A	N/A	N/A	N/A	N/A	N/A	N/A
**Household crowding**	Ideal	N/A	N/A	N/A	N/A	N/A	N/A	Ref	Ref	Ref
	Crowded	N/A	N/A	N/A	N/A	N/A	N/A	1.16 [0.54, 2.44]	1.23 [0.81, 1.89]	1.33 [1.03, 1.69]
	Underoccupied	N/A	N/A	N/A	N/A	N/A	N/A	0.88 [0.41, 1.89]	0.9 [0.72, 1.11]	0.81 [0.70, 0.92]
**Household composition**	Adults-only households	Ref	Ref	Ref	Ref	N/A	N/A	Ref	Ref	Ref
	Households with children	1.04 [0.93, 1.18]	1.18 [0.99, 1.39]	0.68 [0.24, 2.04]	0.88 [0.37, 2.27]	N/A	N/A	1.14 [0.50, 2.56]	1.12 [0.88, 1.43]	0.96 [0.84, 1.09]
	Single households (living alone)	1.39 [1.12, 1.69]	1.61 [1.27, 2.08]	1.16 [0.34, 4.00]	2.17 [0.63, 7.69]	N/A	N/A	2.33 [0.89, 5.88]	1.61 [1.3, 2.00]	1.33 [1.18, 1.52]
**Dwelling type**	House	N/A	N/A	Ref	Ref	N/A	N/A	Ref	Ref	Ref
	Apartment	N/A	N/A	1.72 [0.75, 4.00]	1.49 [0.79, 2.78]	N/A	N/A	1.00 [0.43, 2.33]	0.79 [0.62, 1.02]	0.88 [0.75, 1.02]
**Urbanicity**	Urban	Ref	Ref	Ref	Ref	N/A	N/A	Ref	Ref	Ref
	Semi-urban	0.95 [0.85, 1.06]	1.05 [0.96, 1.16]	0,78 [0.24, 2.17]	0.68 [0.22, 1.79]	N/A	N/A	0.91 [0.44, 1.85]	0.86 [0.70, 1.05]	1.14 [0.98, 1.30]
	Rural	1.02 [0.93, 1.11]	0.97 [0.89, 1.06]	0.38 [0.05, 1.61]	1.41 [0.57, 3.23]	N/A	N/A	1.72 [0.72, 4.17]	0.65 [0.51, 0.83]	0.87 [0.76, 1.00]
