# Supplementary material for: Housing environment and mental health of Europeans during the COVID-19 pandemic: a cross-country comparison
Source: Sci Rep. 2022 Apr 4;12:5612. doi: 10.1038/s41598-022-09316-4 (PMC8978496; doi:10.1038/s41598-022-09316-4)
Supplement: Supplementary file 8 — Supplementary Table S5. [file 41598_2022_9316_MOESM8_ESM.pdf]

Supplemental Table 5. Odds ratio of reporting severe loneliness / severe anxiety / low life satisfaction compared to the reference values in the four cohorts (N=69,136). Model 3.

LONELINESS										
**Exposure**		**DNBC**		**TEMPO**		**Constances**		**UCL Covid-19 Social Study**		
		**Young people**	**Women**	**Men**	**Women**	**Men**	**Women**	**Young people**	**Men**	**Women**
**Access to outdoor facilities**	Yes	Ref	Ref	Ref	Ref	Ref	Ref	Ref	Ref	Ref
	No	1.17 [0.90,1.53]	3.59 [1.56,8.28]	0.14 [0.02, 1.01]	0.46 [0.09, 2.47]	1.25 [1.03,1.51]	1.23 [1.04,1.46]	2.41 [1.17,4.97]	1.10 [0.82,1.48]	1.02 [0.84,1.25]
**Household density**	<43 m2	Ref	Ref	N/A	N/A	Ref	Ref	N/A	N/A	N/A
	≥43 m2	0.89 [0.79,1.00]	1.14 [0.97,1.35]	N/A	N/A	1.36 [1.18,1.58]	1.35 [1.11,1.65]	N/A	N/A	N/A
**Household crowding**	Ideal	N/A	N/A	N/A	N/A	Ref	Ref	Ref	Ref	Ref
	Crowded	N/A	N/A	N/A	N/A	1.41 [1.08,1.84]	1.28 [0.83,1.73]	0.42 [0.21, 0.84]	1.07 [0.71,.160]	1.25 [0.94,1.67]
	Underoccupied	N/A	N/A	N/A	N/A	0.67 [0.57,0.79]	0.86 [0.72,1.04]	0.76 [0.39,1.47]	0.82 [0.63,1.05]	0.91 [0.79,1.05]
**Household composition**	Adults-only households	Ref	Ref	Ref	Ref	Ref	Ref	Ref	Ref	Ref
	Households with children	0.91 [0.80,1.03]	0.88 [0.74,1.04]	0.82 [0.31, 2.23]	1.58 [0.74, 3.57]	1.05 [0.86,1.27]	1.20 [0.93,1.55]	2.87 [1.39,5.92]	0.98 [0.67,1.20]	1.05 [0.91,1.21]
	Single households (living alone)	1.69 [1.38,2.06]	2.33 [1.81,3.00]	0.72 [0.23, 2.26]	5.4 [1.65, 18.1]	8.71 [7.51,10.10]	6.99 [5.69,8.58]	1.58 [0.63,3.96]	3.62 [2.86,4.59]	2.72 [2.33,3.18]
**Dwelling type**	House	N/A	N/A	Ref	Ref	Ref	Ref	Ref	Ref	Ref
	Apartment	N/A	N/A	0.16 [0.02, 1.24]	0.23 [0.04, 1.32]	1.71 [1.46,1.99]	1.50 [1.32,1.70]	0.69 [0.29,1.63]	0.89 [0.67,1.19]	0.98 [0.82,1.17]
**Urbanicity**	Urban	Ref	Ref	Ref	Ref	Ref	Ref	Ref	Ref	Ref
	Semi-urban	0.98 [0.83,1.17]	1.07 [0.98,1.17]	0.34 [0.11, 0.99]	0.7 [0.31, 1.55]	N/A	N/A	1.28 [0.67,2.43]	1.13 [0.91,1.41]	1.02 [0.90,1.16]
	Rural	0.97 [0.86,1.10]	1.05 [0.96,1.13]	0.34 [0.08, 1.2	0.77 [0.34, 1.65]	1.05 [0.89,1.26]	1.14 [0.91,1.42]	2.43 [1.65,5.09]	1.11 [0.84,1.47]	0.94 [0.81,1.09]
ANXIETY										
**Exposure**		**DNBC**		**TEMPO**		**Constances**		**UCL Covid-19 Social Study**		
		**Young people**	**Women**	**Men**	**Women**	**Men**	**Women**	**Young people**	**Men**	**Women**
**Access to outdoor facilities**	Yes	Ref	Ref	N/A	N/A	Ref	Ref	Ref	Ref	Ref
	No	0.99 [0.75,1.30]	1.35 [0.69,2.62]	N/A	N/A	1.21 [0.92, 1.59]	1.12 [0.89, 1.41]	1.21 [0.53,2.79]	1.02 [0.63,1.64]	1.03 [0.81,1.31]
**Household density**	<43 m2	Ref	Ref	N/A	N/A	Ref	Ref	N/A	N/A	N/A
	≥43 m2	0.76 [0.67,0.86]	1.07 [0.91,1.26]	N/A	N/A	1.02 [0.84, 1.24]	0.86 [0.75,0.99]	N/A	N/A	N/A
**Household crowding**	Ideal	N/A	N/A	N/A	N/A	Ref	Ref	Ref	Ref	Ref
	Crowded	N/A	N/A	N/A	N/A	1.16 [0.79,1.70]	1.09 [0.82,1.44]	1.89 [0.81,4,40]	0.98 [0.45,2.12]	0.95 [0.68,1.32]
	Underoccupied	N/A	N/A	N/A	N/A	0.86 [0.69,1.07]	0.78 [0.68,0.91]	2.36 [1.08,5.19]	0.62 [0.41,0.94]	0.82 [0.68,0.98]
**Household composition**	Adults-only households	Ref	Ref	N/A	N/A	Ref	Ref	Ref	Ref	Ref
	Households with children	0.94 [0.83,1.06]	1.07 [0.90,1.27]	N/A	N/A	1.18 [0.93,1.48]	1.13 [0.97,1.33]	0.42 [0.16,1.05]	0.81 [0.51,1.28]	1.10 [0.91,1.34]
	Single households (living alone)	1.23 [1.00,1.51]	1.05 [0.82,1.35]	N/A	N/A	1.55 [1.23,1.95]	0.91 [0.76,1.10]	1.02 [0.35,2.96]	0.39 [0.26,0.58]	0.48 [0.39,0.58]
**Dwelling type**	House	N/A	N/A	N/A	N/A	Ref	Ref	Ref	Ref	Ref
	Apartment	N/A	N/A	N/A	N/A	1.18 [0.95,1.47]	0.82 [0.70,0.96]	0.41 [0.16,1.02]	0.92 [0.57,1.48]	1.08 [0.87,1.34]
	Urban	Ref	Ref	N/A	N/A	Ref	Ref	Ref	Ref	Ref

**Urbanicity**	Semi-urban	0.87 [0.75,1.01]	1.03 [0.94,1.13]	N/A	N/A	N/A	N/A	0.59 [0.28,1.23]	1.29 [0.85,1.97]	0.95 [0.79,1.13]
	Rural	0.98 [0.84,1.14]	1.03 [0.93,1.15]	N/A	N/A	0.83 [0.64,1.06]	1.02 [0.87,1.19]	0.56 [0.21,1.48]	1.38 [0.83,2.30]	0.91 [0.74,1.12]

LIFE SATISFACTION

**Exposure**		**DNBC**		**TEMPO**		**Constances**		**UCL Covid-19 Social Study**		
		**Young people**	**Women**	**Men**	**Women**	**Men**	**Women**	**Young people**	**Men**	**Women**
**Access to outdoor facilities**	Yes	Ref	Ref	Ref	Ref	N/A	N/A	Ref	Ref	Ref
	No	1.16 [0.88, 1.54]	1.12 [0.55, 2.27]	0.12 [0.01, 1.28]	0.32 [0.05, 2.78]	N/A	N/A	0.83 [0.36, 1.92]	1.3 [1, 1.69]	1.22 [1.01, 1.45]
**Household density**	<43 m2	Ref	Ref	N/A	N/A	N/A	N/A	N/A	N/A	N/A
	≥43 m2	0.92 [0.81, 1.04]	0.81 [0.65, 1.01]	N/A	N/A	N/A	N/A	N/A	N/A	N/A
**Household crowding**	Ideal	N/A	N/A	N/A	N/A	N/A	N/A	Ref	Ref	Ref
	Crowded	N/A	N/A	N/A	N/A	N/A	N/A	1.56 [0.7, 3.45]	1.16 [0.74, 1.82]	1.25 [0.95, 1.64]
	Underoccupied	N/A	N/A	N/A	N/A	N/A	N/A	0.87 [0.39, 1.92]	1.09 [0.85, 1.37]	0.87 [0.75, 1]
**Household composition**	Adults-only households	Ref	Ref	Ref	Ref	N/A	N/A	Ref	Ref	Ref
	Households with children	1.02 [0.9, 1.16]	1.16 [0.98, 1.37]	0.88 [0.29, 2.94]	0.93 [0.39, 2.44]	N/A	N/A	0.99 [0.42, 2.33]	1.22 [0.95, 1.59]	0.81 [0.68, 0.97]
	Single households (living alone)	1.35 [1.1, 1.67]	1.64 [1.28, 2.13]	0.98 [0.27, 3.45]	2.08 [0.58, 7.69]	N/A	N/A	1.92 [0.64, 5.88]	1.12 [0.88, 1.43]	1.05 [0.92, 1.2]
**Dwelling type**	House	N/A	N/A	Ref	Ref	N/A	N/A	Ref	Ref	Ref
	Apartment	N/A	N/A	0.2 [0.02, 2.17]	0.45 [0.07, 4.17]	N/A	N/A	1.35 [0.57, 3.13]	0.83 [0.63, 1.11]	0.85 [0.72, 1.01]
**Urbanicity**	Urban	Ref	Ref	Ref	Ref	N/A	N/A	Ref	Ref	Ref
	Semi-urban	0.95 [0.81, 1.11]	1.04 [0.94, 1.14]	0.87 [0.23, 2.86]	0.77 [0.23, 2.13]	N/A	N/A	1.02 [0.48, 2.17]	0.78 [0.63, 0.96]	1.14 [0.98, 1.33]
	Rural	0.96 [0.85, 1.1]	0.98 [0.9, 1.06]	0.4 [0.05, 1.89]	1.54 [0.61, 0.79]	N/A	N/A	1.35 [0.57, 3.23]	0.57 [0.43, 0.75]	0.91 [0.78, 1.05]
